# E2F/Rb Family Proteins Mediate Interferon Induced Repression of Adenovirus Immediate Early Transcription to Promote Persistent Viral Infection

**DOI:** 10.1371/journal.ppat.1005415

**Published:** 2016-01-25

**Authors:** Yueting Zheng, Thomas Stamminger, Patrick Hearing

**Affiliations:** 1 Department of Molecular Genetics and Microbiology, School of Medicine, Stony Brook University, Stony Brook, New York, United States of America; 2 Institute for Clinical and Molecular Virology, University of Erlangen-Nuremberg, Erlangen, Germany; University of Michigan, UNITED STATES

## Abstract

Interferons (IFNs) are cytokines that have pleiotropic effects and play important roles in innate and adaptive immunity. IFNs have broad antiviral properties and function by different mechanisms. IFNs fail to inhibit wild-type Adenovirus (Ad) replication in established cancer cell lines. In this study, we analyzed the effects of IFNs on Ad replication in normal human cells. Our data demonstrate that both IFNα and IFNγ blocked wild-type Ad5 replication in primary human bronchial epithelial cells (NHBEC) and TERT-immortalized normal human diploid fibroblasts (HDF-TERT). IFNs inhibited the replication of divergent adenoviruses. The inhibition of Ad5 replication by IFNα and IFNγ is the consequence of repression of transcription of the E1A immediate early gene product. Both IFNα and IFNγ impede the association of the transactivator GABP with the E1A enhancer region during the early phase of infection. The repression of E1A expression by IFNs requires a conserved E2F binding site in the E1A enhancer, and IFNs increased the enrichment of the E2F-associated pocket proteins, Rb and p107, at the E1A enhancer *in vivo*. PD0332991 (Pabociclib), a specific CDK4/6 inhibitor, dephosphoryles pocket proteins to promote their interaction with E2Fs and inhibited wild-type Ad5 replication dependent on the conserved E2F binding site. Consistent with this result, expression of the small E1A oncoprotein, which abrogates E2F/pocket protein interactions, rescued Ad replication in the presence of IFNα or IFNγ. Finally, we established a persistent Ad infection model *in vitro* and demonstrated that IFNγ suppresses productive Ad replication in a manner dependent on the E2F binding site in the E1A enhancer. This is the first study that probes the molecular basis of persistent adenovirus infection and reveals a novel mechanism by which adenoviruses utilize IFN signaling to suppress lytic virus replication and to promote persistent infection.

## Author Summary

Interferons play important roles in both innate and adaptive immunity, and have broad antiviral properties. We demonstrate that type I (IFNα) and type II (IFNγ) IFNs inhibit the replication of divergent adenoviruses via an evolutionally conserved E2F binding site. IFNs augment the association of the tumor suppressors Rb and p107 with the E1A enhancer region *in vivo* to repress viral immediate early transcription. By comparing the properties of wild type and E2F site mutant viruses, we show that the IFN–E2F/Rb axis is critical for restriction of adenovirus replication to promote persistent viral infection. Relief of E2F/Rb repression counteracts IFN signaling whereas enforcement of E2F/Rb interaction mimics IFN signaling. These results reveal a novel mechanism by which adenoviruses utilize IFN signaling to suppress lytic virus replication and promote persistent infection.

## Introduction

Interferons (IFNs) are widely expressed cytokines that have pleiotropic effects on cells. IFNs play important roles in both innate and adaptive immunity [1,2]. There are three types of IFNs: I, II and III. Type I IFNs (α, β, ε, κ and ω) are produced by multiple cell types following the activation of pathogen pattern recognitions receptors and function in both an autocrine and paracrine manner. Type II IFN (γ) is produced by T cells and natural killer cells, as well as other cells of the immune system. Type III IFNs (λs) play an important role in mucosal cell immunity. All three types of IFNs bind to cell surface receptors that activate Janus kinases to phosphoryate STAT (Signal Tranducer of Activated Transcription) proteins [1,2]. STAT proteins homo- and heterodimerize and induce the expression of numerous IFN-stimulated genes (ISGs) that have antimicrobial properties [3]. IFNs have broad antiviral properties and function by different mechanisms.

Adenoviruses (Ad) are ubiquitous pathogens infecting a wide range of vertebrates. Ad infection is generally associated with mild respiratory, ocular, and gastrointestinal diseases, but Ads have been recognized in recent years as significant pathogens in immunocompromised patients [4]. IFNs fail to inhibit wild-type Ad replication in established cancer cell lines [5–7]. The resistance of wild-type Ad to the effects of IFNs is due to multiple counteracting effects of viral gene products. The Ad E1A proteins block IFN signaling by binding STAT proteins and preventing the activation of interferon-stimulated gene factors 3 (ISGF3) complex by type I IFNs and IFNγ activation factor (GAF) complex by type II IFN [8]. The E1A proteins also bind and disrupt the hBre1 transcription complex and prevent IFN-induced histone H2B monoubiquitination and associated ISG expression [9,10]. Both actions of E1A lead to the global suppression of ISG expression. Analogously, the Ad E1B-55K protein inhibits the expression of cellular ISGs through its transcriptional repression domain [11,12]. Numerous studies have shown that promyelocytic leukemia nuclear bodies (PML-NB) play an important role in cellular intrinsic and IFN-induced antiviral immunity [13]. The Ad E4-ORF3 protein antagonizes the functions of PML-NB by disrupting these structures and sequestering antiviral components including PML and Daxx [7,14]. The Ad E1B-55K:E4-ORF6 ubiquitin ligase complex also targets Daxx for proteasome degradation [15]. Finally, Ad VA RNA-I inactivates PKR to prevent IFN-induced phosphorylation of the eIF2α translation factor which inhibits global protein translation during the late phase of viral infection [6].

Current models of interplay between Ad infection and IFN signaling have mostly been conducted in cancer cell lines. Such cells are coupled with abnormal signal transduction, unlimited proliferation, and evasion of apoptosis, and are compromised in many normal signaling pathways. Indeed, it has been shown that the Ad E1B-55K protein was able to inhibit a set of ISG expression in response to type I IFN signaling in primary human cells, which has not been reported in established cell lines [12]. Moreover, a recent study showed wild-type Ad exhibits an enhanced virus load in the organs of the STAT2-knockout Syrian hamsters compared to wild-type animals, revealing an important role of type I IFNs in controlling Ad replication *in vivo* [16]. To understand Ad pathogenesis in a natural context, this study focused on understanding the regulation of Ad replication by IFNs in normal human cells.

The E1A protein is the first Ad product expressed following infection and it is indispensible for virus growth [17]. In addition to antagonizing IFN signaling, the E1A proteins directly interact with a number of cellular proteins to regulate viral and cellular gene expression, and promote cell cycle progression [17]. E1A gene expression is regulated by an upstream enhancer region primarily via the activity of the cellular transcription factor GA-binding protein (GABP)[18–21]. As a tetrameric transcriptional complex, GABP (also known as E4TF-1 and NRF-2) is composed of two GABPα subunits which bind DNA, and two GABPβ subunits which transactivate gene expression [22,23]. Deletion of the two GABP binding sites in the E1A enhancer region dramatically decreases E1A expression [19,20]. A separate E1A enhancer segment, located between the GABP binding sites, regulates transcription from the entire Ad genome [21]. Finally, both GABP binding sites in the E1A enhancer are located adjacent to sequences that bind E2F transcription factors, although these E2F sites are dispensable for E1A expression [19,21].

In this study, we analyzed the effects of IFNs on Ad replication in normal human cells. Our data demonstrate that both IFN‹ and IFNγ blocked the replication of divergent adenoviruses in these cells. The inhibition of Ad5 replication by IFN‹ and IFNγ is the consequence of repression of transcription of the E1A immediate early gene product. The repression of E1A expression by IFNs is associated with the binding of E2F/Rb complexes to a conserved E2F site in the E1A enhancer. Finally, we established an Ad persistent infection model *in vitro* and demonstrated that IFNγ supresses productive Ad replication in a manner dependent on the conserved E2F binding site in the E1A enhancer. These results reveal a novel mechanism by which adenoviruses utilize IFN signaling to inhibit virus replication and to promote persistent infection.

## Results

### IFNα and IFNγ inhibit wild type Ad5 replication in normal human cells

**Fig 1:**
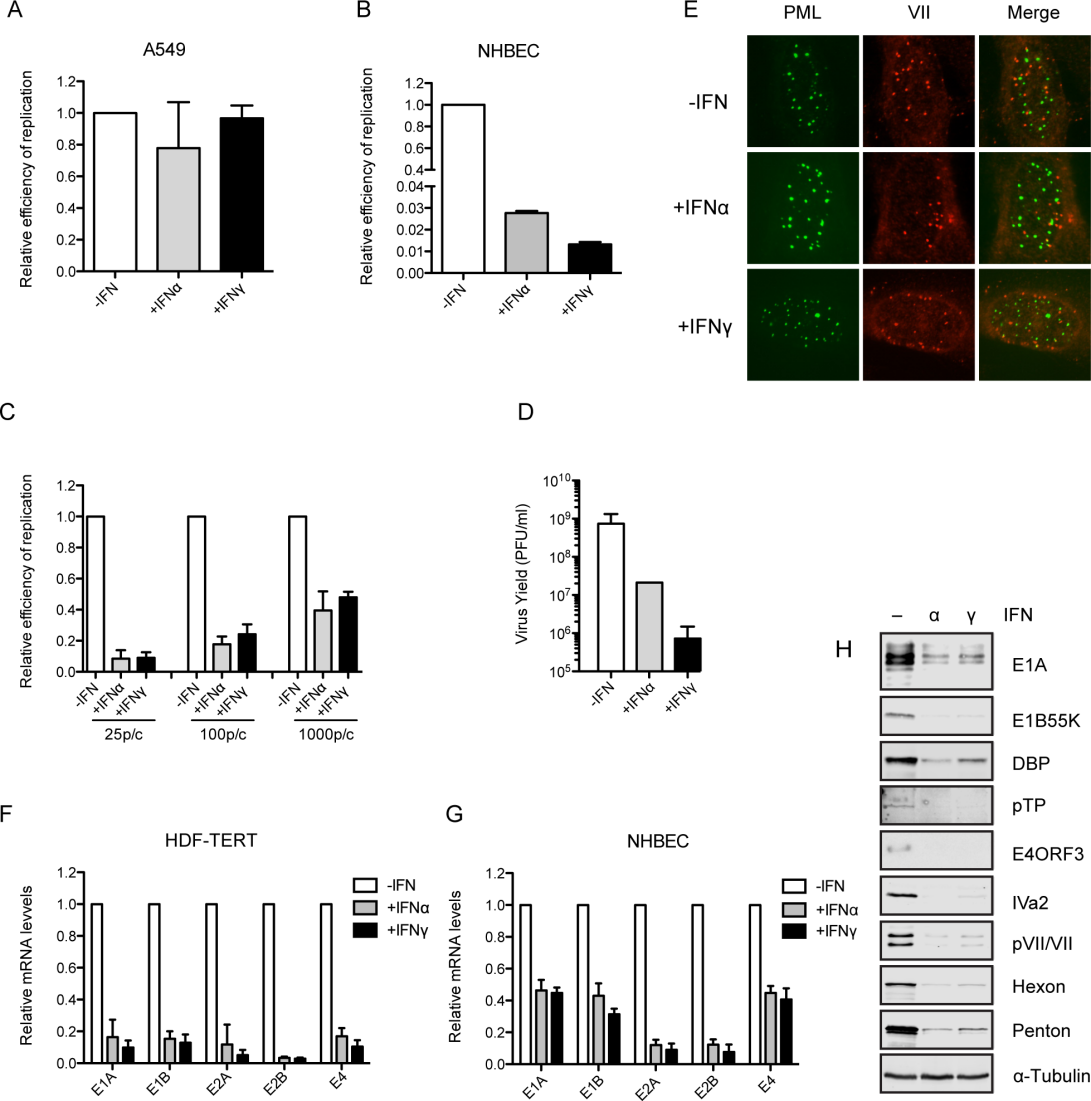
IFNα and IFNγ inhibit adenovirus replication and early gene expression in normal human cells. (A–C) Wild-type Ad5 replication was evaluated in multiple cells plus or minus IFN treatment. A549 (A) and NHBEC (B) cells were pretreated with IFNα, IFNγ or left untreated for 24 hr, and then infected with phenotypically wild-type Ad5 *dl*309 at 100 virus particles/cell. Cells were harvested at 2 and 24 hr post-infection and viral DNA input (2 hr) and viral DNA replication (24 hr) was quantified by qPCR. HDF-TERT cells (C) were pretreated with IFNs or left untreated for 24 hr, and then infected with *dl*309 at 25, 100 or 1,000 virus particles/cell. Viral DNA replication was quantified at 48 hr post-infection by qPCR. The data are plotted as mean ± sd, n = 3. (D) HDF-TERT cells were pretreated with IFNs or left untreated for 24 hr and then infected with *dl*309 at 25 virus particles/cell. Infectious virus yields were measured at 6 d post-infection by plaque assay on A549 cells. The data are plotted as mean ± sd, n = 3. (E) HDF-TERT cells were pretreated with IFNs or left untreated for 24 hr and then infected with *dl*309 at 1,000 particles/cell and immunostained for PML (green) and the Ad major core protein VII (red) at 6 hr post-infection. Merged images are shown in the right column. (F) HDF-TERT cells HDF-TERT cells were pretreated with IFNs or left untreated for 24 hr and then infected with *dl*309 at 25 virus particles/cell. Ad early mRNA levels were quantified by RT-qPCR with RNA samples isolated at 48 hr post-infection. The results were normalized to GAPDH mRNA levels and the fold-change in Ad early gene expression compared to untreated cells is plotted as mean ± sd, n = 3. (G) NHBEC cells were pretreated with IFNs or left untreated for 24 hr and then infected with wild-type Ad5 at 100 virus particles/cell and Ad early gene mRNA levels were quantified at 24 hr post-infection as described in (F). n = 2. (H) HDF-TERT cells HDF-TERT cells were pretreated with IFNs or left untreated for 24 hr and then infected with *dl*309 at 25 virus particles/cell. Ad proteins were analyzed by Western blot using specific antiera directed against early (E1A, E1B55K, DBP, pTP, E4ORF3), intermediate (Iva2) and late gene products (pVII/VII, Hexon, Penton). α-tubulin is shown in the bottom panel as a loading control for the samples.

To evaluate Ad replication in normal human cells during an IFN response, primary human bronchial epithelial cells (NHBEC) were used, in comparison to the established epithelial adenocarcinoma cell line A549 (Fig 1A and 1B). Strikingly, Ad5 replication in NHBEC was inhibited by IFNα and IFNγ 40-fold and 70-fold, respectively. Neither IFNα nor IFNγ was able to block replication of Ad5 in A549 cells. NHBEC only survive for a few passages in culture and vary from lot to lot. Therefore, we sought other primary human cells where IFNs inhibit Ad replication. We utilized a normal, non-transformed human diploid fibroblast cell line immortalized by human telomerase (HDF-TERT) that is permissive for Ad infection. Ad5 had an infectious particle/PFU ratio of ~1000:1 in HDF-TERT cells compared to ~20:1 in A549 cells due to reduced infectivity of HDF-TERT cells. We established the time line of the Ad5 life cycle in HDF-TERT cells. The majority of incoming viral genomes (detected by immunofluorescence using an antibody against the viral core protein VII) were still in the cytoplasm at 2 hr post-infection; complete entry of viral genomes into the nucleus required ~ 6 hr (S1A Fig). By 16 hr post-infection, the number of core protein VII foci had significantly declined indicating viral early gene transcription had occurred [24]. Analyses of Ad immediate early (E1A) and early mRNA levels (E1B, E2A, E2B and E4) showed an exponential increase in early gene expression from 6 to 24 hr post-infection (S1B Fig). The kinetics of viral DNA replication was determined (S1C Fig); viral DNA replication began during the 18–24 hr period and increased substantially thereafter with a 4-log total increase in genome copy number. Viral early gene (E4-ORF3) and intermediate gene (IVa2) expression was evident as DNA replication occurred and late gene products continued to accumulate through 144 hr post-infection (S1D and S1E Fig). There was no cytopathic effect observed up to 6 days post-infection even at a multiplicity of infection (MOI) of 1000 virus particles/cell where nearly all cells were infected.

To evaluate Ad5 replication in HDF-TERT cells in the presence or absence of IFNs, cells were incubated with IFNα, IFNγ or left untreated for 24 hr, followed by Ad5 infection at low MOI (25 virus particles/cell). Viral DNA replication was quantified at 48 hr post-infection (Fig 1C). Consistent with the results observed in NHBEC, both IFNα and IFNγ significantly inhibited virus replication (20- and 50-fold, respectively). The effect of IFNs on Ad replication was moderately diminished at higher MOIs but Ad replication was still inhibited in IFN-treated cells. IFNα and IFNγ reduced infectious virus production 2 and 3 logs, respectively (Fig 1D). IFNs did not block the entry of viral genomes into the nucleus and the majority of viral genomes did not colocalize with PML-NB with or without IFN treatment (Fig 1E and S1F Fig). We examined Ad5 early mRNA levels with or without IFN treatment. Ad immediate early (E1A) and early gene expression (E1B, E2A, E2B and E4) was significantly suppressed in IFN-treated cells compared to untreated cells (Fig 1F). Similar results were observed in NHBEC (Fig 1G). The reduction in viral mRNA levels correlated with decreased early, intermediate and late gene expression (Fig 1H).

### IFNs inhibit Ad replication by repressing immediate early gene expression

**Fig 2:**
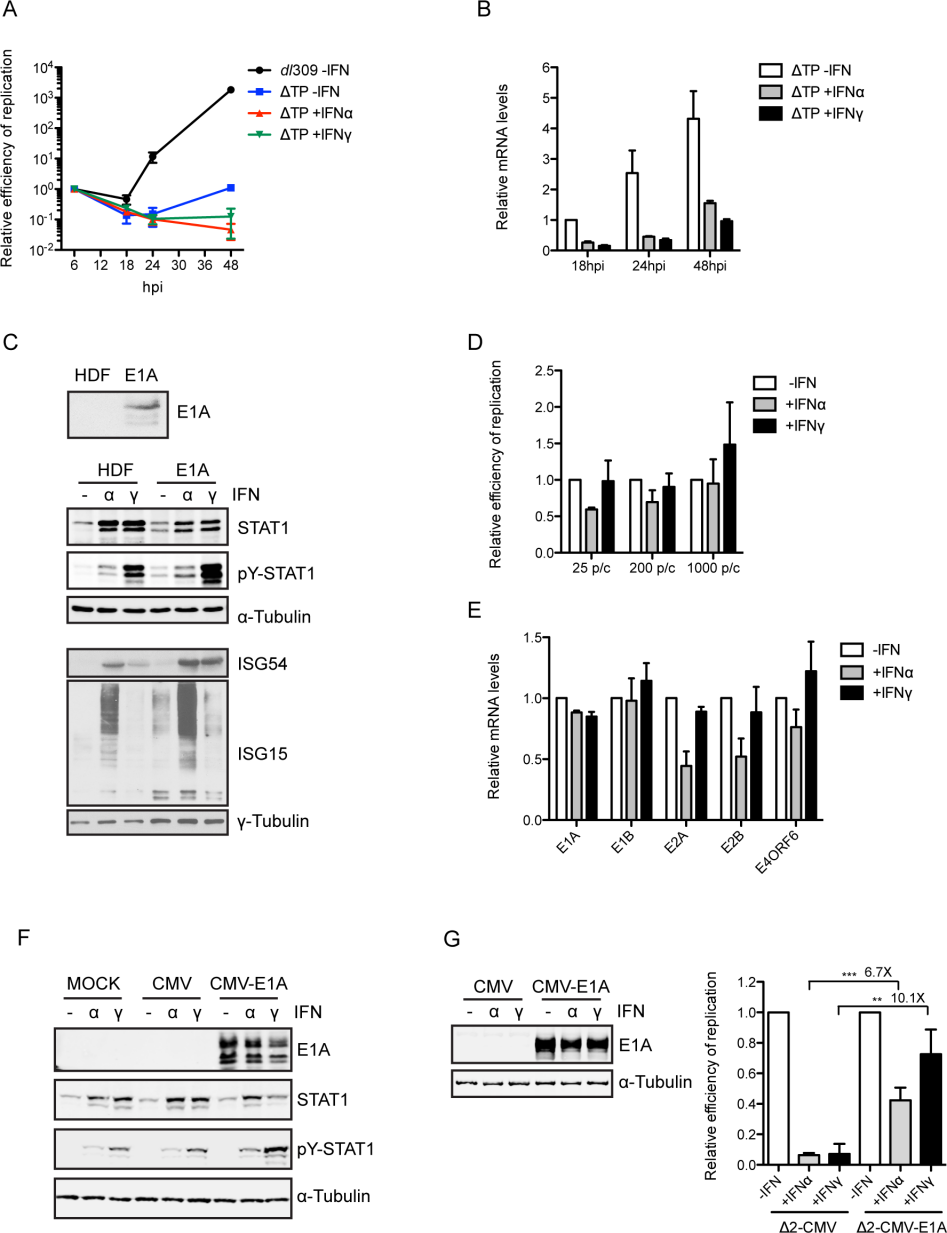
Inhibition of Ad replication by IFNs is blocked by E1A expression. (A) HDF-TERT cells treated with IFNs or left untreated for 24 hr and then infected with *dl*309 or the replication-defective mutant virus ΔTP-GFP at 25 virus particles/cell. Nuclear DNA was isolated at the indicated time points and viral DNA levels were quantified by qPCR. (B) HDF-TERT cells treated with IFNs or left untreated for 24 hr and then infected with ΔTP-GFP at 25 virus particles/cell. E1A mRNA levels were quantified by RT-qPCR. The results were normalized to cellular GAPDH mRNA levels. The data are plotted as mean ± sd, n = 3. (C) HDF-TERT-E1A cells treated with IFNs or left untreated for 24 hr and Western blot analyses were performed at 24 hr post-infection to detect expression and phosphorylation of STAT1, and the expression of ISG54 and ISG15. E1A 12S and 13S products are shown in the top panel. γ-tubulin is shown in the bottom panel as a loading control for the samples. (D) HDF-TERT-E1A cells treated with IFNs or left untreated for 24 hr and then infected with *dl*309 at 25, 200 or 1000 virus particles/cell. Nuclear DNA was isolated at 48 hr post-infection and viral DNA levels were quantified by qPCR. The values were normalized to 1.0 in untreated cells and are plotted as mean ± sd, n = 3. (E) HDF-TERT-E1A cells treated with IFNs or left untreated for 24 hr and then infected with *dl*309 at 25 virus particles/cell. E1A mRNA levels were quantified at 24 hr post-infection by RT-qPCR. The values were normalized to 1.0 in untreated cells and are plotted as mean ± sd, n = 3. (F) HDF-TERT were infected with *in*340-Δ2-CMV (Ad-CMV) or *in*340-Δ2-CMV-E1A (Ad-CMV-E1A) viruses at 5,000 virus particles/cell for 1 hr and subsequently treated with IFNs or left untreated. Cellular extracts were prepared at 24 hr post-treatment and analyzed by Western blot using antibodies against E1A, STAT1 and STAT1 (pY701). α-tubulin is shown in the bottom panel as a loading control for the samples. (G) HDF-TERT cells were infected and treated as in (F) and super-infected with *dl*309 at 200 virus particles/cell 24 hr after the addition of IFNs. *dl*309 DNA replication was quantified 48 hr post-infection by qPCR using a *dl*309-specific primer pair. The values were normalized to 1.0 in untreated cells and are plotted as mean ± sd, n = 3. (** P ≤ 0.01, *** P ≤ 0.001).

IFNs may repress early gene expression directly or IFNs may inhibit viral DNA replication resulting in a corresponding decrease in early gene expression. To examine these possibilities, HDF-TERT cells were infected with a replication-defective mutant virus ΔTP-GFP. ΔTP-GFP contains a disruption in the coding sequences for Ad5 terminal protein, a protein that is absolutely essential for viral DNA replication [25]. Even though the relative copy number of ΔTP-GFP viral genomes in the presence and absence of IFNs did not increase, E1A mRNA levels were reduced by both IFNα and IFNγ throughout the time course of the experiment (Fig 2A and 2B). These results demonstrate that IFNs inhibit Ad early gene expression in a replication-independent manner. E1A is the first viral protein expressed after infection and it is essential for efficient activation of all Ad gene expression [17]. It is possible that IFNs suppress E1A transcription directly, resulting in the observed phenotype, rather than having a global effect on Ad early gene expression. To test this hypothesis, we generated an E1A-expressing HDF-TERT cell line (HDF-TERT-E1A); the major E1A isoforms were expressed in these cells (Fig 2C, top panel) at levels similar to that found in HDF-TERT cells infected with Ad5 (S2A Fig, lanes 2 and 3). Since E1A can inhibit IFN signaling [8], we examined IFN signaling in HDF-TERT-E1A cells (Fig 2C). E1A expression did not impede IFN signaling in these cells; increases in STAT1 protein levels and STAT1 phosphorylation as well as the induction of IGS54 and ISG15 expression were observed in HDF-TERT-E1A cells following IFNα and IFNγ treatment. Ad DNA replication and early gene expression were almost completely restored in HDF-TERT-E1A cells infected at low MOI and treated with IFNs (Fig 2D and 2E). The levels of all Ad early mRNAs were completely restored in HDF-TERT-E1A cells infected at high MOI and treated with IFNα or IFNγ (S2C Fig).

As a viral oncogene, E1A is able to transform cells [17]. Stable expression of E1A might lead to transformation of HDF-TERT losing the biological property of normal human cells. We evaluated Ad replication following IFN treatment when E1A was only transiently expressed. We generated an Ad expression vector (*in*340-Δ2-CMV) which contains the CMV promoter/enhancer in place of the E1A enhancer region and E1A/E1B coding sequences. The complete E1A coding region was inserted downstream of the CMV promoter. HDF-TERT cells were infected with the Ad-CMV-E1A virus or the control virus for 1 hr, followed by the addition of IFNα or IFNγ. Twenty four hr later, cells extracts were prepared and E1A expression was analyzed, or the cells were super-infected with Ad5 and Ad5 replication was assayed at 48 hr post-infection. Neither IFNα nor IFNγ reduced E1A expression in cells infected with the Ad-CMV-E1A virus (Fig 2G, left). Ad5 replication was significantly reduced in IFN-treated cells coinfected with the control Ad-CMV virus. Consistent with the results obtained using HDF-TERT-E1A cells, ectopic expression of E1A partially rescued this defect in IFN-treated cells (Fig 2G, right). As observed with HDF-TERT-E1A cells, transient expression of E1A did not impair IFN signaling in these assays (Fig 2F) even though the E1A proteins were significantly over-expressed compared to infection with Ad5 (S2A Fig, lanes 3 and 4). Collectively, these results demonstrate that both IFNα and IFNγ inhibit Ad replication by repressing E1A gene expression.

### IFNs block recruitment of GABP to the E1A enhancer region

**Fig 3:**
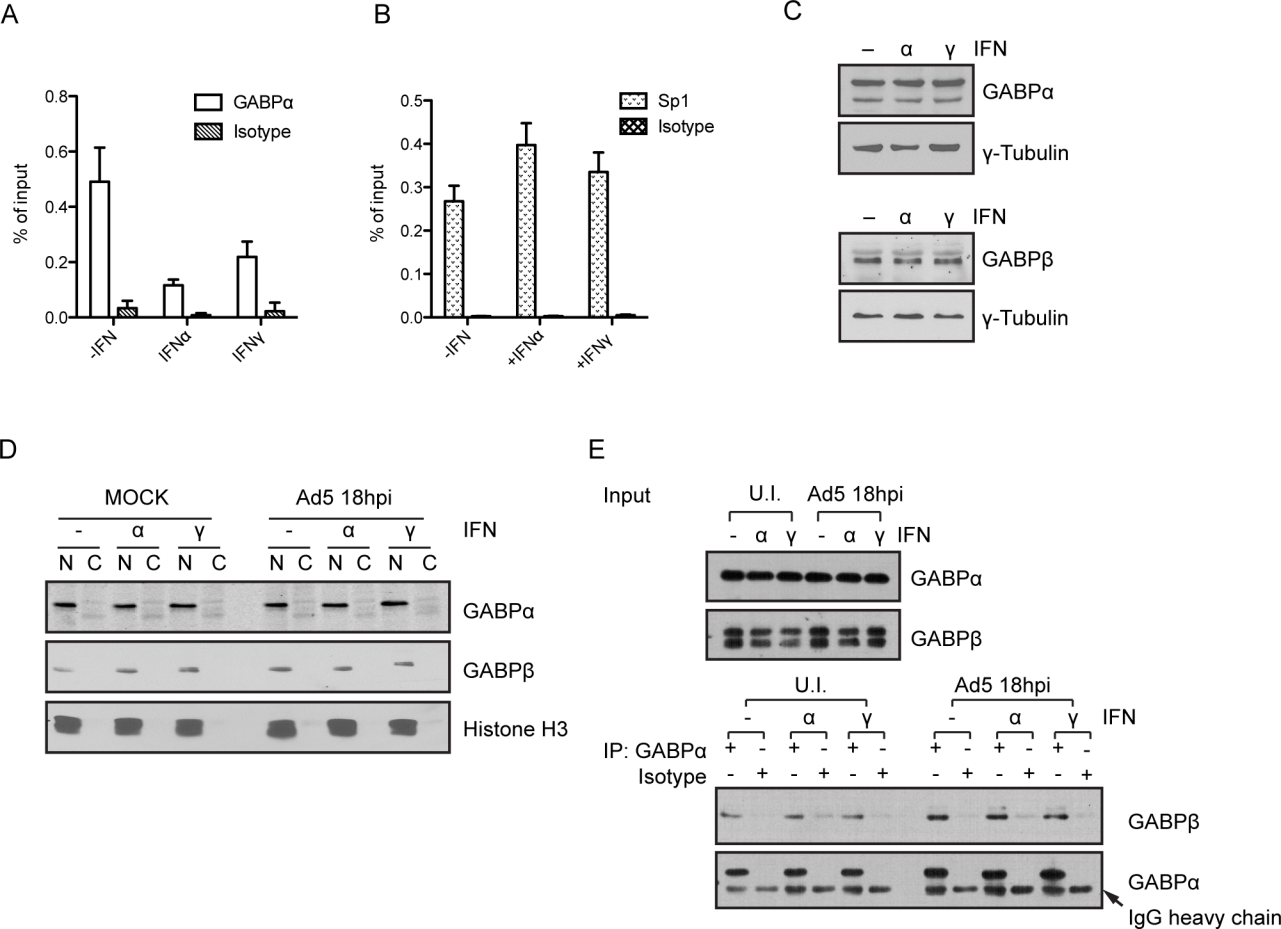
IFNs inhibit the association of transcription factor GABP with the E1A enhancer region. (A and B) HDF-TERT cells were treated with IFNs or left untreated for 24 hr and then infected with *dl*309 at 200 virus particles/cell. ChIP assays were performed with samples isolated at 18 hr post-infection using an antibody directed against GABPα or an isotype-matched control (A), or using an antibody directed against Sp1 or an isotype-matched control (B). Immunoprecipitated DNA was quantified by qPCR and the enrichment is represented as percentage of input. The values are plotted as mean ± sd, n = 3. (C) HDF-TERT cells were treated with IFNs or left untreated. Cellular extracts were prepared at 24 hr post-treatment and analyzed by Western blot using antibodies directed against GABPα and GABPβ. γ-tubulin was used as a loading control for the samples. (D) HDF-TERT cells were treated with IFNs or left untreated for 24 hr and then infected with *dl*309 200 virus particles/cell or mock-infected. Nuclear (N) and cytoplasmic (C) fractions were prepared 18 hr post-infection. Western blot analyses were performed using antibodies against GABPα and GABPβ. Histone H3 was used a nuclear marker to verify the subcellular fractionation. (E) IFN treatment and infection of HDF-TERT cells was carried out as described in (D). Total cell extracts were isolated at 18 hr post-infection and used for immunoprecipitation (IP) with an antibody against GABPα or an isotype-matched control. Immunocomplexes were analyzed for GABPβ coIP by Western blot. The top panel shows GABPα and GABPβ protein levels in cell lysates (Input). The bottom panel shows the results of the IPs.

The cellular transcription factor GABP binds to two sites in the E1A enhancer region and GABP is the major regulator of E1A transcription [18,19,21]. These two sites have synergistic effects on E1A transcription; when both sites are deleted, E1A expression is dramatically diminished. Therefore, we examined the interaction of GABP with the E1A enhancer region *in vivo* by ChIP. At 18 hr post-infection, prior to the onset of viral DNA replication (S1C Fig), IFNα or IFNγ decreased the association of GABP with the E1A enhancer *in vivo* 3- and 2.5-fold, respectively (Fig 3A). Expression of both GABPα and GABP β in IFN-treated cells was the same as that found in untreated cells (Fig 3C). In HDF-TERT cells, GABPα and β were primarily nuclear localized and their localization were not altered by either IFNα or IFNγ (Fig 3D). Finally, we performed coimmunoprecipitation assays to determine if IFNs impaired endogenous GABPα:GABP β interaction. These results did not reveal any differences in IFN-treated cells (Fig 3E).

The cellular transcription factor Sp1 binds to several sites in the Ad5 inverted terminal repeat (ITR)[26] that are adjacent to the E1A enhancer region. We analyzed if Sp1 binding to the ITR was altered by IFN signaling *in vivo*. Sp1 bound to the Ad5 ITR at similar levels in the presence and absence of IFNs (Fig 3B). This result suggests that reduced binding of GABP to the E1A enhancer following IFN treatment is target specific and not due to reduced global accessibility of the left-end of the Ad5 genome.

### IFN signaling inhibits E1A expression via a conserved *cis*-acting repressor element

**Fig 4:**
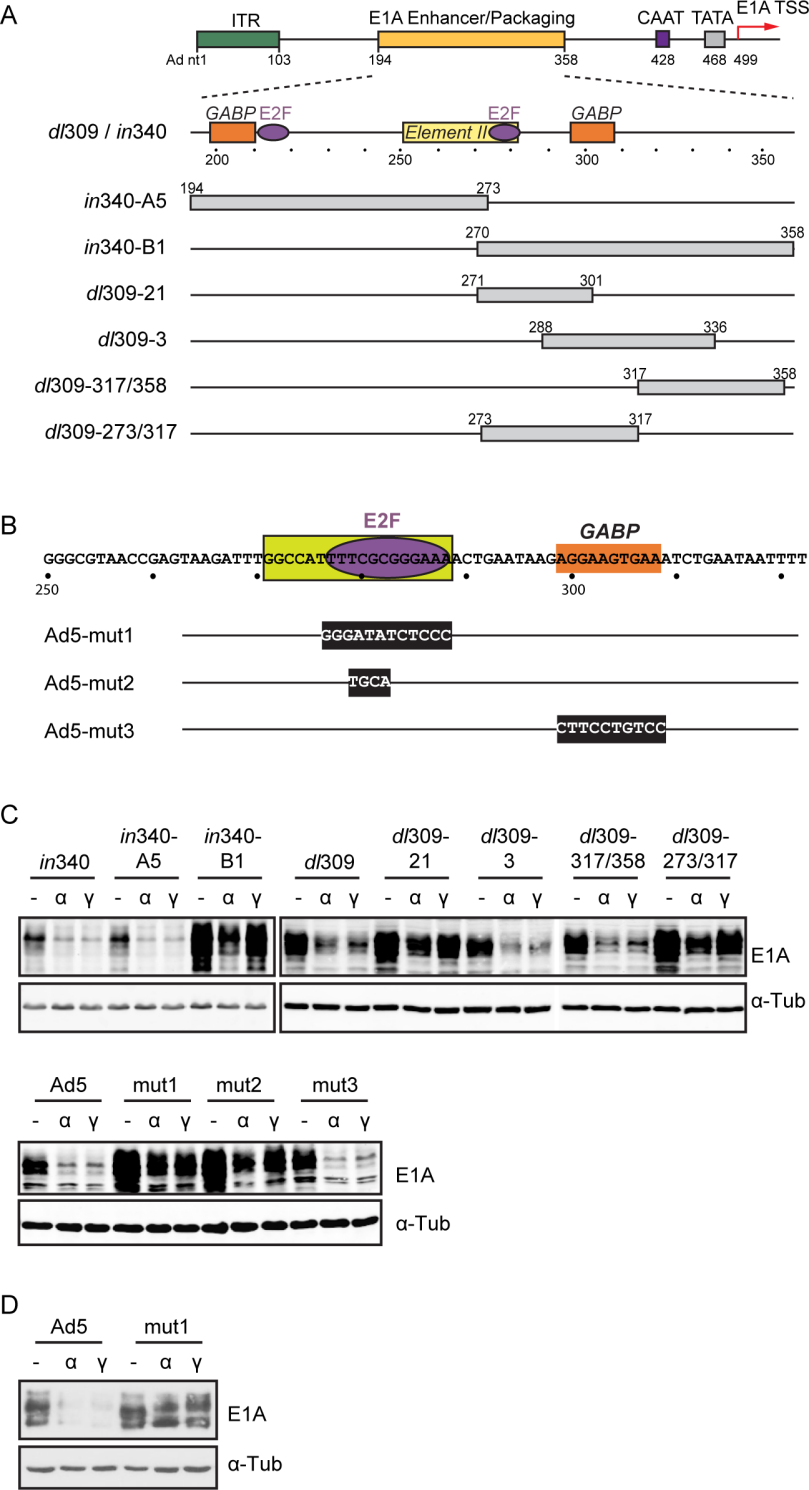
The E1A enhancer region contains a repressor element that responds to IFNs. (A) Schematic view of the left end of the Ad5 genome is shown at the top including the inverted terminal repeat (ITR), E1A enhancer region, CAAT and TATA boxes, and transcription start site (TSS). Shown below are the GABP and E2F binding sites in the E1A enhancer and the boundaries of enhancer element II. E1A enhancer region deletion mutants are depicted below the schematics. (B) Sequence of Ad5 nt 250–320 including the E2F and GABP binding sites and flanking sequences and the location of linker scanning mutants generated in this region (Ad5-mut1–mut3). The black rectangles with letters indicate nucleotide substitutions. The yellow rectangle indicates the boundaries of the IFN-induced repressor site delimited by the deletions mutants in (A). (C) HDF-TERT cells were treated with IFNs or left untreated for 24 hr and then infected with the mutant viruses illustrated in (A) and (B), as well as the corresponding parent virus (*dl*309, *in*340 and Ad5-WT). E1A protein levels were examined at 48 hr post-infection by Western blot. α-tubulin was used as a loading control for the samples. (D) NHBEC cells were pretreated with IFNs or left untreated for 24 hr and then infected with Ad5-WT at 100 virus particles/cell and E1A protein levels were analyzed at 24 hr post-infection by Western blot. α-Tubulin was used as a loading control.

We hypothesized that IFNs may induce the binding of a transcriptional repressor to the E1A promoter/enhancer region. We examined the effect of IFNs on E1A expression using a panel of existing [20,21,27] and newly created E1A enhancer region mutants (Fig 4A and 4B). E1A expression of deletion mutant *in*340-B1, but not of the adjacent deletion mutant *in*340-A5, was completely restored in IFNγ-treated cells, and partially restored in IFNα-treated cells (Fig 4C). This finding indicated that an IFN-induced repressor binding site is located in the downstream half of the E1A enhancer region, corresponding to Ad5 nt 270–358. We also noted that basal E1A expression with mutant *in*340-B1 was significantly augmented compared to the parent virus *in*340. We next screened a set of mutants carrying smaller deletions within the *in*340-B1 interval of the E1A enhancer. Interestingly, *dl*309-21 (Δ271–301) and *dl*309-Δ273/317 exhibited significantly increased basal E1A protein levels and were refractory to IFN-mediated repression. In contrast, E1A expression with the adjacent mutants *dl*309-3 (Δ288–336) and *dl*309-Δ317/358 was still fully repressed by IFN treatment (Fig 4C). We conclude that Ad5 sequences located within nt 271–288 are the target of IFN-mediated repression of E1A expression.

Mutations were introduced into the core of the nt 271–288 interval (Ad5-mut1, Ad5-mut2) and in the adjacent GABP binding site (Ad5-mut3)(Fig 4B). E2F/Rb family proteins bind to sequences positioned at nt 280 (TTTCGCGGGAAA) *in vitro* [18,28]. Ad5-mut1 disrupts this entire sequence and Ad5-mut2 disrupts the core of the E2F binding site (CGCG). Ad5-mut1 and Ad5-mut2 exhibited efficient E1A expression in the presence of IFNs compared with Ad5-WT (Fig 4C). Basal E1A expression was increased with both mutants minus IFN, and this reflected increased levels of E1A mRNAs in infected cells (S2B Fig). Efficient E1A expression with Ad5-mut1 in the presence of IFNs also was observed in NHBEC (Fig 4D). Ad5-mut3 had normal basal expression levels and E1A expression was fully repressed by IFNs. The compelling insensitivity of Ad5-mut2 to IFNα and IFNγ strongly suggests that E2F/Rb family protein binding to the IFN-induced repressor site mediates suppression of E1A expression.

**Fig 5:**
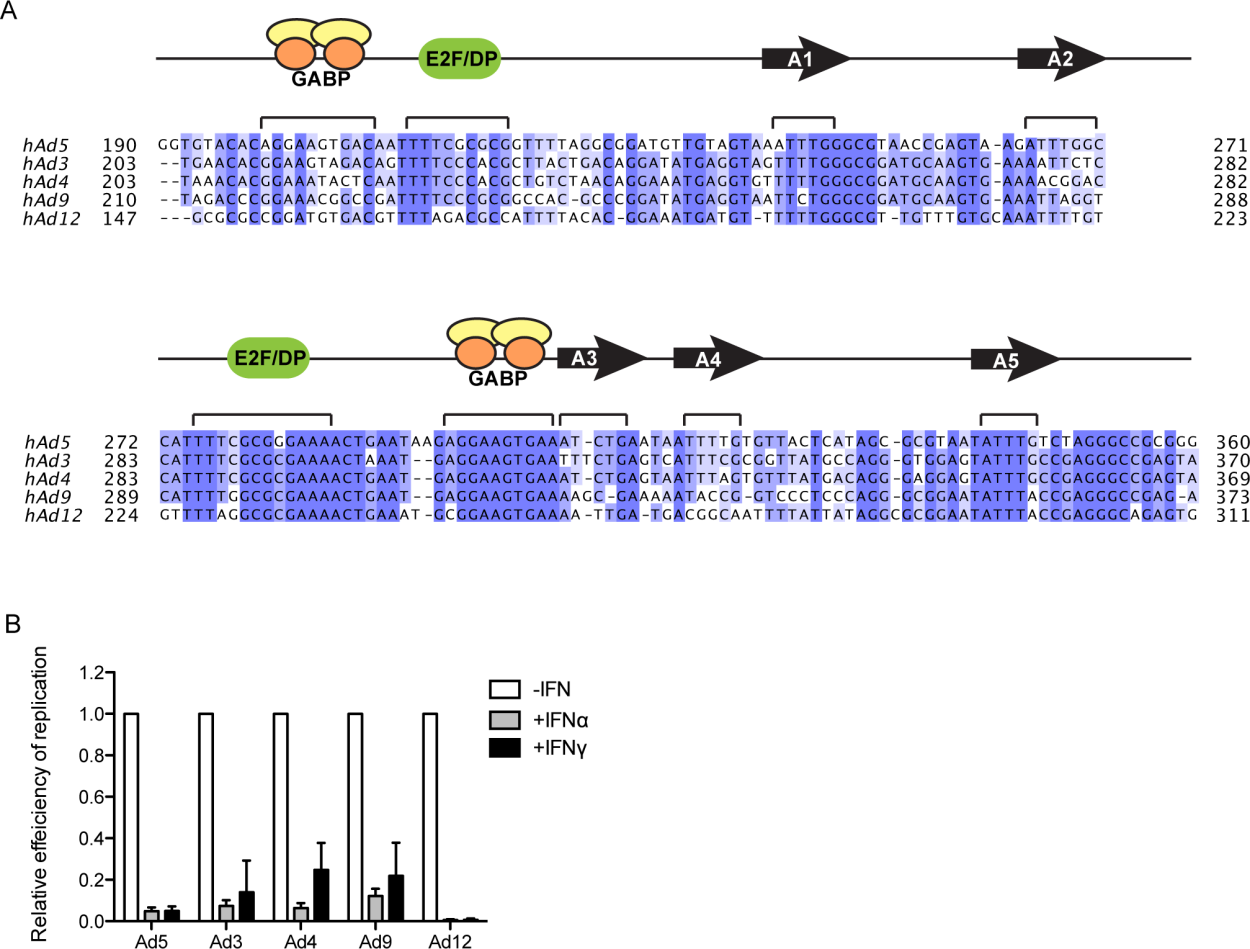
IFN inhibition of replication is conserved among different Ad serotypes. (A) Nucleotide sequence alignment of the E1A enhancer regions from different Ad subgroups (serotypes Ad5, Ad3, Ad4, Ad9 and Ad12 corresponding to Ad subgroups C, B, E, D and A, respectively). Homologies (≥75% identity) are shaded in blue. The GABP and E2F binding sites, as well packaging repeats (arrowhead A1-5), are indicated. (B) HDF-TERT cells were treated with IFNs or left untreated for 24 hr and then infected with 25 virus particles/cell Ad5, Ad3, Ad4, or Ad12 or 5 virus particles/cell Ad9. Nuclear DNA was isolated at 6 hr post-infection to determine virus input and at 72 hr post-infection with Ad3, Ad4, Ad9 and Ad12 or at 48 hr post-infection with Ad5 to measure replication. Viral DNA levels were quantified by qPCR using a primer that recognizes conserved sequence in E1A (AdE1A-Pan) in combination with a serotype-specific primer (S1 Table in S1 Materials and Methods). The values were normalized to 1.0 in untreated cells and are plotted as mean ± sd, n = 3.

DNA sequence alignment of the E1A enhancer regions of divergent Ad serotypes revealed that the E2F binding site located between nt 270–290 is highly conserved (Fig 5A). Thus, we hypothesized that IFNs would repress viral replication of evolutionarily divergent Ads. IFN-treated and untreated HDF-TERT cells were infected with Ad3, Ad4, Ad5, Ad9, and Ad12 (subgroups B, E, C, D and A, respectively) at MOIs that resulted in similar levels of infection. DNA replication of all of these Ad serotypes was inhibited by IFNα and IFNγ treatment (Fig 5B).

### E2F/Rb family proteins associate with the E1A repressor site *in vitro* and *in vivo*

**Fig 6:**
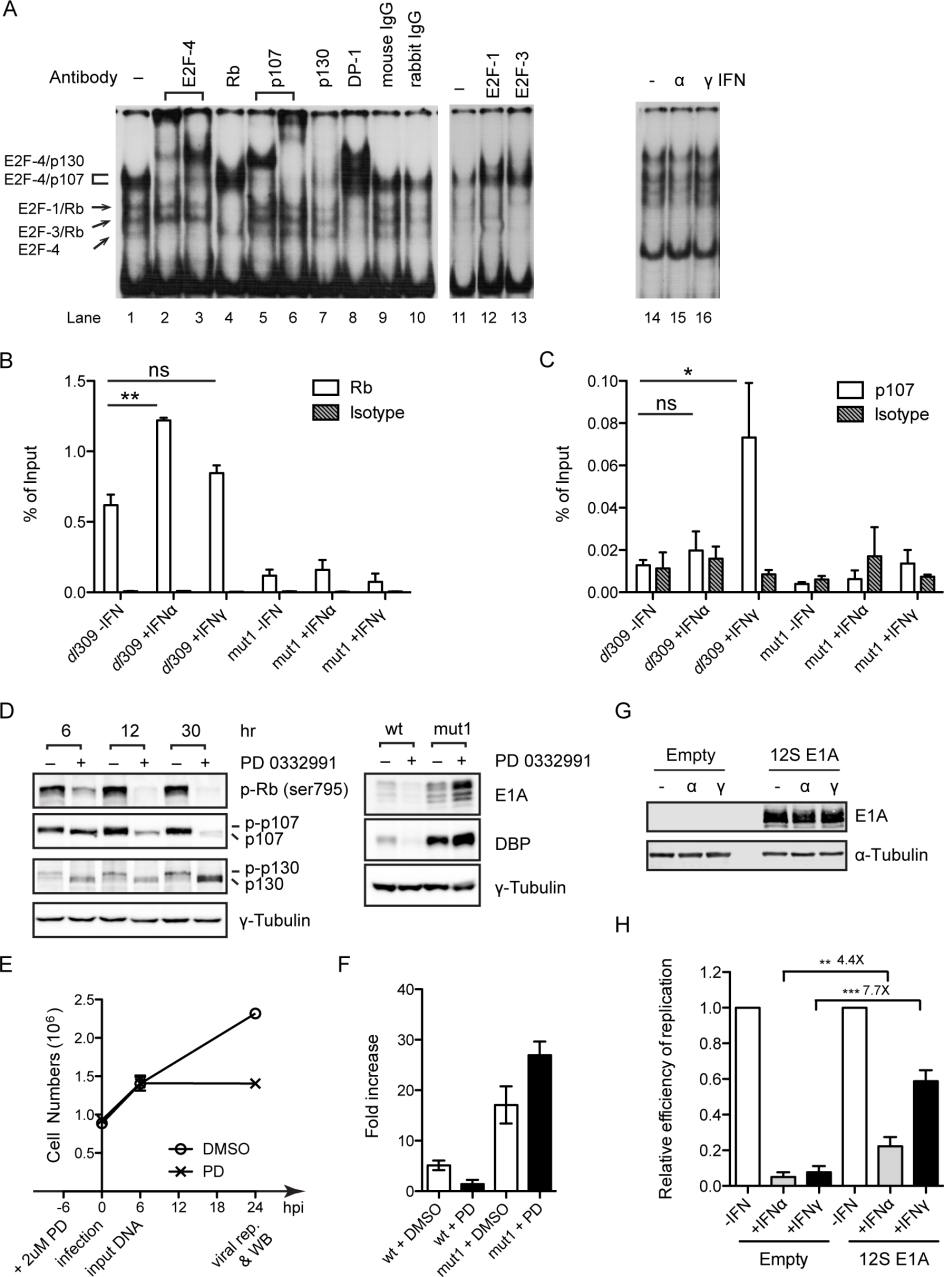
Role of E2F-Rb family proteins in the inhibition of E1A expression by IFNs. (A) EMSA binding reactions were performed using nuclear extract from HDF-TERT cells and a ^32^P-labeled probe corresponding to the IFN-regulated E1A enhancer repressor element (E1A-ENH, Ad5 nt 271–288, lane 1). Lanes 2–10 show EMSA binding reactions that contained specific antibodies directed E2F, DP, and Rb family members (E2Fs 1, 3 and 4, Rb, p107, p130, and DP1) or control antibodies (normal mouse and rabbit). The compositions of DNA-protein EMSA complexes are indicated on the left. Lanes 11–13 show binding reactions with HDF-TERT nuclear extracts prepared from untreated cells or cells treated with IFNα or IFNγ for 24 hr. (B and C) ChIP assays were performed to examine the binding of Rb and p107 to the E1A enhancer region *in vivo* with or without IFN treatment. HDF-TERT cells were treated with IFNs or left untreated for 24 hr and then infected with 200 virus particles/cell *dl*309 or Ad5-mut1. ChIP assays were performed at 18 hr post-infection using antibodies against Rb (B), p107 (C), with isotype-matched controls. Immunoprecipitated DNAs were quantified by qPCR (primer pairs are listed in S1 Table in S1 Materials and Methods). Enrichment is represented as percentage of input DNA and the values represent the mean ± sd, n = 3. (* P ≤ 0.05, ** P ≤ 0.01). (D–F) The effect of the cdk4 inhibitor PD0332991 on Ad5 replication via the E2F binding site in the E1A enhancer region was evaluated. HDF-TERT cells were incubated with 2 μM PD0332991 or DMSO for 6 hr prior to virus infection. (D, left) Cell extracts were prepared at 6, 12 and 30 hr following PD0332991 pretreatment and Rb, p107 and p130 phosphorylation was examined by Western blot. (D, right) PD0332991 pretreated cells were infected with Ad5-WT and Ad5-mut1 at 200 virus particles/cell and Ad early gene expression (E1A, DBP) was analyzed by Western blot at 24 hr post-infection. γ-tubulin was used as a loading control for the samples. (E) Cell numbers were determined after 6 hr PD0332991 pretreatment (0), and in virus-infected cells at 6 and 24 hr post-infection. (F) Cells were pretreated with PD0332991 for 6 hr and infected as described in (D) and viral DNA replication was quantified at 24 hr post-infection by qPCR. The values were normalized to the input at 6 hr post-infection and are plotted as mean ± sd. n = 3. (G and H) HDF-TERT cells were infected Ad-CMV-12S-E1A or the empty control virus, Ad-CMV, at 5,000 virus particles/cell for 1 hr and the cells were left untreated or treated with IFNs for 24 hr. The cells were super-infected with *dl*309 at 200 virus particles/cell. (G) Cells were harvested at 48 hr post-infection and E1A expression was analyzed by Western blot. α-tubulin was used as a loading control for the samples. (H) Cells were harvested at 48 hr post-infection and viral DNA replication was quantified by qPCR using a *dl*309-specific primer pair (see S1 Table in S1 Materials and Methods). The values were normalized to 1.0 in untreated cells and are plotted as mean ± sd, n = 3. (** P ≤ 0.01, *** P ≤ 0.001).

The E2F/Rb pathway is disrupted in nearly all human cancers [29] and all previous analyses of the E1A enhancer region were conducted using nuclear extracts prepared from established cancer cell lines [18,28]. Thus, we examined E2F binding to the E1A repressor element using HDF-TERT cells. Nuclear extract was prepared from HDF-TERT cells and analyzed by EMSA using a radiolabeled probe corresponding to the IFN-induced E1A repressor site (Ad nt 271 to 288)(Fig 6A). Multiple DNA-protein complexes were observed that were identified using specific antibodies to E2F/Rb family proteins. The fastest migrating complex contained E2F-4 and DP-1 while slower migrating complexes contained E2F3/DP1/Rb and E2F1/DP1/Rb (lanes 2–4, 8, 12 and 13). The complexes with the slowest migration contained E2F-4/DP1 with p107 or p130 (lanes 5–8). The specificity of each antibody was ensured by competition using the corresponding peptides used to generate the antibodies. Identical complexes were observed using nuclear extracts from IFN-treated HDF-TERT cells (Fig 6A, lanes 14–16).

We performed ChIP experiments to evaluate the enrichment of Rb family proteins at the E1A enhancer early after infection with or without IFN treatment (Fig 6B and 6C). These results showed a 2-fold increase in Rb binding following IFNα treatment with no significant effect of IFNγ, and a 5-fold increase in p107 binding following IFNγ treatment with no significant effect of IFNα. The binding of Rb and p107 to the E1A enhancer of Ad5-mut1 was reduced 4-fold compared to wild-type Ad5 without IFN treatment, and no significant increase in binding of either of these proteins to Ad5-mut1 was observed following IFN treatment (Fig 6B and 6C). These results demonstrate that IFN treatment induces the binding of E2F/Rb complexes to the E1A enhancer. The reduction in Rb and p107 binding to near background levels with Ad5-mut1 suggests that E2F family proteins primarily associate with the downstream E2F site centered at the IFN-induced repressor site rather that at the upstream E2F binding site. This conclusion is consistent with the E1A enhancer mutant screen where a mutant in the upstream E2F binding site showed repression of E1A expression following IFN treatment (Fig 4, *dl*309-A5).

PD0332991 (Palbociclib) is a specific CDK4/6 inhibitor and can cause cell cycle arrest through dephosphorylation of Rb family proteins [30]. To avoid changes in Rb family protein phosphorylation by contact inhibition, low density HDF-TERT cells were incubated with PD0332991 and Rb, p107 and p130 phosphorylation was evaluated. Rb, p107 and p130 phosphorylation was reduced within 6 hr of PD0332991 addition (Fig 6D, left, 0 hpi) although growth arrest was not evident until later times (Fig 6E). PD0332991 inhibited Ad5-WT replication whereas Ad5-mut1 replication was increased (Fig 6F); these results correlated with E1A and E2A DNA binding protein (DBP) expression levels (Fig 6D, right). Thus, PD0332991 treatment mimicked the effects of IFNs. It is well established that the E1A 12S protein can directly interact with Rb family proteins, leading to their dissociation from E2Fs and activation of E2F target gene expression [17]. Ectopic expression of the E1A12S protein partially rescued Ad5 replication in the presence of IFNs (Fig 6H). IFN treatment did not reduce E1A 12S protein expression with the vector used in these experiments (Fig 6G); the E1A 12S protein was over-expressed compared to cells infected with Ad5 (S2A Fig, lanes 3 and 5). Collectively, these experiments reveal that IFNs inhibit Ad replication and E1A expression via E2F/Rb family proteins and induction of transcriptional repressor activity.

### PML-NB and IFI16 are not required for IFN-mediated E1A repression

PML nuclear bodies (PML-NB) play important roles in intrinsic and IFN-mediated immunity against various viruses [13]. Three major components of PML-NB are PML, Daxx and Sp100. We previously reported that PML and Daxx, but not Sp100, mediated an IFN-induced antiviral response during Ad infection [14]. Interestingly, it has been shown that PML can induce cellular senescence in an Rb-dependent manner [31–33]. In these studies, PML relocalized Rb and induced heterochromatin formation and silencing of E2F target genes, leading to cell cycle arrest. Thus, we analyzed if PML-NBs participate in repression of Ad replication and E1A expression in HDF-TERT cells following IFN treatment. We generated a series of PML, Daxx and Sp100 knockdown HDF-TERT cell lines using shRNA expression. PML and Sp100 protein levels were completed depleted in the corresponding knockdown cells and Daxx expression was completely depleted in two out of four shDaxx-cells and significantly reduced in the other two lines; normal levels of all three proteins were found in cells expressing control shRNA (S2A Fig, left). Ad5 replication was inhibited by both IFNα and IFNγ in single knockdown cells, as well as in the control cells, despite high MOI infection (S2B Fig, left). RT-qPCR confirmed that E1A mRNA levels were correspondingly reduced (S3C Fig). A recent study revealed that PML, Daxx and Sp100 have synergistic effects on the suppression of herpesvirus replication with greater effects of multiple knockdown compared to single knockdown [34]. We generated HDF-TERT cells in which two or three PML-NB proteins were simultaneously depleted (Daxx plus PML, shDP, PML plus Sp100, shPS, and all three protein, shDPS; S2A Fig, right). IFNs repressed Ad replication in these double- and triple-knockdown cells to the same extent as with parental or control knockdown cells (S2B Fig, right).

IFI16, a HIN-200 family protein, senses intracellular viral DNA leading to IRF3 activation and IFNβ expression [35]. Ectopic expression of IFI16 in prostate cancer cell lines increases p21 expression and inhibits E2F-stimulated transcription [36–38]. Taken together, these results suggested that IFI16 might regulate global E2F transcription activity upon IFN treatment in HDF-TERT cells. We generated three independent pools of IFI16 knockdown cells, shIFI16-1-1, shIFI16-1-2 and shIFI16-3-1 (S3A Fig). Although treatment of the IFI16 knockdown cells with IFNs slightly increased IFI16 protein levels (S3B Fig), Ad5 replication was still inhibited by IFNα and IFNγ treatment to the extent observed in control cells (S3C Fig). We conclude the neither PML-NB or IFI16 are required for IFN-mediated repression of Ad5 replication.

### The IFN-induced repressor element in the E1A enhancer region is important for the establishment of persistent Ad5 infection

**Fig 7:**
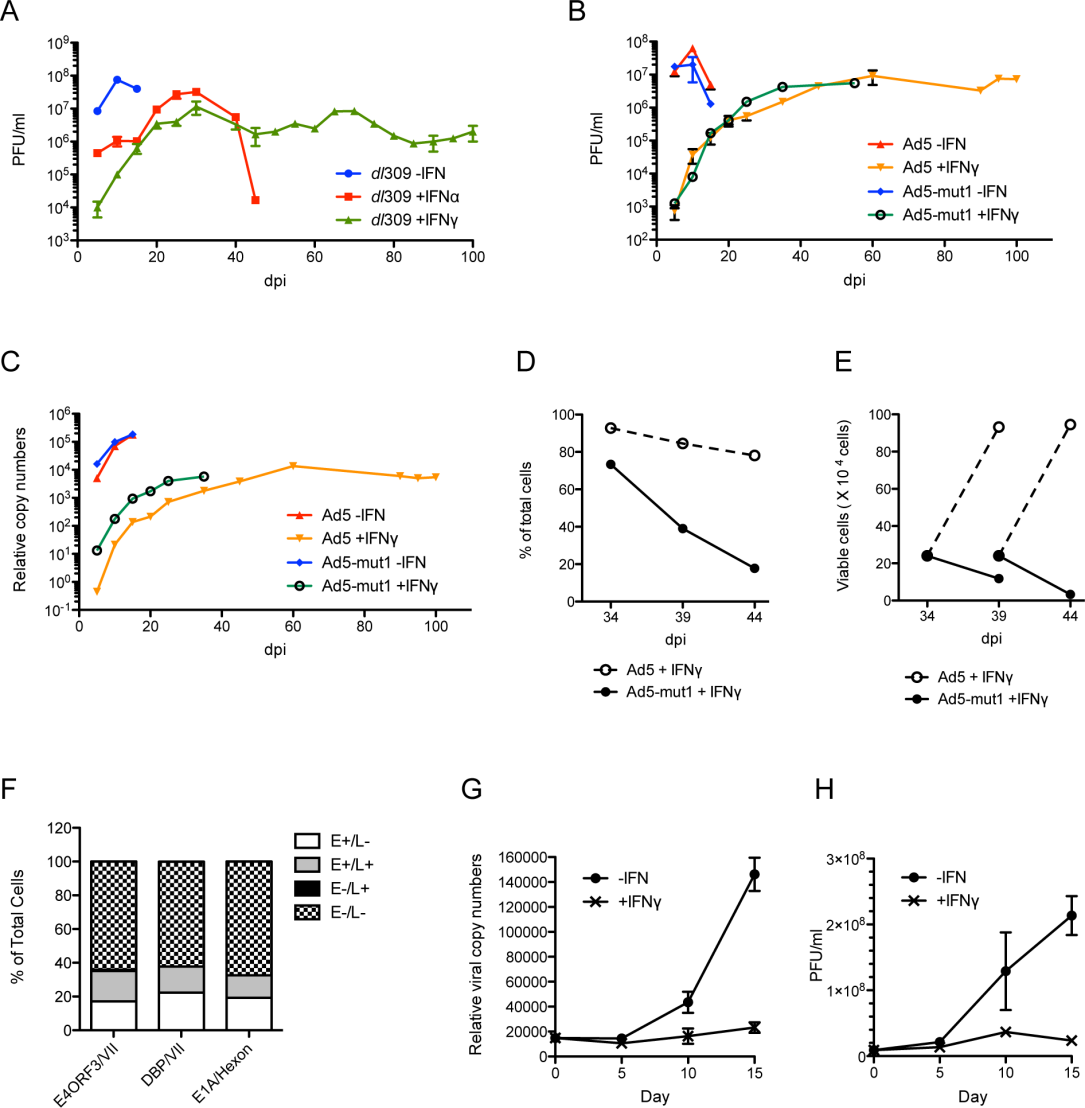
IFNs promote the establishment of persistent Ad infection. (A–C) HDF-TERT cells were infected with *dl*309 (A), Ad5-WT (B) or Ad5-mut1 (C) at 25 virus particles/cell. Infected cells were cultured in the presence and absence of IFNα or IFNγ. Growth media plus or minus IFNs were replenished every 5 days. Periodically, infectious virus yields were determined by plaque assay (A, B) and viral DNA replication was quantified by qPCR (C). (D and E) The viability of infected cells was determined by trypan blue staining at the indicated times, and plotted as a percentage of total cells (D) or the number of total viable cells (E). (F) Immunofluorescence assays were used to determine the percentage of Ad protein-positive cells. At 113 days post-infection, Ad5-infected HDF-TERT cells treated with IFNγ were immunostained for Ad early proteins (E1A, DBP, E4-ORF3) and late proteins (pVII, Hexon). Cells were scored positive or negative for the expression of viral proteins, and the percentages of 250 cells in 5–6 random views were plotted. E and L stand for early and late protein expression, respectively. (G and H) The change from persistent infection to lytic infection upon withdrawal of IFNγ was examined. At 107 days post-infection, Ad5-WT-infected HDF-TERT cells treated with IFNγ (shown in panel B) were seeded into new plates in duplicate. When the cells reached ~80% confluence, IFNγ was removed from one set and IFNγ was maintained in the second set. Cells were subcultured at day 5 and growth medium was replenished at day 10. Ad5 DNA replication was quantified by qPCR (G) and infectious virus yield was determined by plaque assay (H) at days 5, 10 and 15. The plots represent the average of two independent experiments.

Previous studies demonstrated that T lymphocytes in tonsil and adenoid tissues are the primary reservoir for latent Ad [39,40]. As important cytokines mediating innate and adaptive immunity, type I and type II IFNs could inhibit Ad replication and promote the establishment of persistent infection. The conserved IFN-induced repressor element in the E1A enhancer region may be involved in Ad latency by repressing E1A expression in response to IFNs and allow Ad to evade immune surveillance and clearance. To test this hypothesis, HDF-TERT cells were infected with Ad5 at low MOI in the presence or absence of IFNs. Infected cells were cultured for an extended period of time and the production of infectious virus quantified by plaque assay (Fig 7A). During the first 5 days of infection, no cytopathic effect was observed in any infected cells. In the absence of IFNs, peak virus yield was reached at 10 days post-infection (~10^8^ PFU/ml). Cytopathic effect was observed by day 8–10 (S5 Fig,–IFN) and full cytopathic effect was observed at 15 days post-infection. In contrast, in IFN-treated cells, virus yields gradually increased over 25–30 days, giving a peak of 1-2x10^7^ PFU/ml. IFNα postponed the onset of cytopathic effect until 45 days post-infection. In IFNγ-treated cells, no cytopathic effect was observed throughout the entire period (up to 100 days post-infection). These results demonstrate that both IFNα and IFNγ can promote establishment of long-term Ad infection. An intermediate amount of infectious virus was produced throughout the course of infections with IFNγ treatment (10^6^–10^7^ PFU/ml) indicating the establishment of persistent, not latent, infection.

We examined the role of the E2F binding site in the E1A enhancer region in an IFN response during long-term infection of HDF-TERT cells. HDF-TERT cells were infected with Ad5-WT or Ad5-mut1 in the presence of absence of IFNγ. Both infectious virus yield and viral genome copy number were measured during the course of infection (Fig 7B and 7C). In the absence of IFN treatment, both Ad5-WT and Ad5-mut1 reached peak virus production at 10 days post-infection, and full cytopathic effect was observed at 15 days post-infection. In IFNγ-treated cells, virus production of Ad5-WT and Ad5-mut1 gradually increased from 0 to 30 days post-infection. Cells infected with Ad5-mut1 exhibited cytopathic effect from days 30 through 55 when the experiment was terminated, whereas Ad5-WT infection continued for 100 days with minimal cell death (Fig 7B and S5 Fig). Viral genome copy number correlated well with infectious virus yields throughout the course of these infections (Fig 7C). In the presence of IFNγ, Ad5-mut1-infected cells showed reduced cell viability and proliferation from approximately day 30 onward in comparison to Ad5-WT-infected cells (Fig 7D and 7E) as well as enhanced cytopathic effect (S5A Fig, +IFNγ). As expected, Ad5-mut1 also showed enhanced E1A expression compared to Ad5-WT of the course of infection (S5B Fig). IFNγ-treated cells infected with Ad5-WT were analyzed by immunofluorescence at 113 days post-infection to examine viral early and late gene expression in individual cells (Fig 7F). These result showed that ~40% of the infected cells were positive for viral gene expression with some cells expressing only early proteins and other cells expressing both early and late proteins.

We also examined the fate of long-term infected cells upon the withdrawal of IFNγ (Fig 7G and 7H), There were no significant changes in viral genome copy number and infectious virus yield 5 days after IFNγ removal compared to IFNγ-treated cells. However, significant increases in viral genome copy number and infectious virus yield were observed by 10 and 15 days following IFNγ removal; full cytopathic effect was observed by day 15. Collectively, we conclude that IFNγ treatment represses the Ad5 lytic cycle in infected HDF-TERT cells which promotes persistent infection, and that this effect requires the E2F binding site in the E1A enhancer region.

## Discussion

Adenoviruses establish two different types of infection in the host. Primary infection occurs in epithelial cells, e.g., the nasopharyngeal mucosa with Ad5, resulting in lytic infection and the production of progeny virus. Following acute infection, Ad5 establishes latent infection in the mucosa-associated lymphoid tissue, preferentially T-lymphocytes in tonsil and adenoid tissues [40,41]. Cellular mechanisms that regulate lytic Ad infection are well understood, but the molecular basis for the control of persistent Ad infections was not understood. Here, we demonstrate that both type I and type II IFN signaling leads to the repression of Ad5 immediate early gene expression in normal human cells. This reduction in E1A expression leads to suppression of all subsequent aspects of the virus life cycle. Our results show that repression of E1A expression by IFNs requires a conserved E2F binding site in the E1A enhancer region. E2Fs can transcriptionally activate or repress gene expression depending on their interactions with Rb family proteins [42]. IFNs augmented the binding of the tumor supressors Rb and p107, well characterized E2F binding partners and transcriptional repressors [42], to the E1A enhancer region *in vivo*. Mutation of the conserved E2F binding site in the E1A enhancer abrogated the effects of IFNs on E1A expression and Ad replication. Collectively, our results demonstrate that the IFN–E2F/Rb axis is critical for restriction of adenovirus replication during type I and type II IFN responses.

Given the negative role that the E2F binding site in the Ad5 E1A enhancer region has on both basal and IFN-regulated immediate early gene expression (Fig 5), it was surprising that this E2F site is conserved across divergent Ad serotypes. Indeed, both IFNα and IFNγ treatment of cells repressed the replication of Ads in five evolutionarily distinct subgroups. These observations suggested that Ads may utilize the conserved E2F site in the E1A enhancer to suppress E1A expression in certain infection contexts, for example during persistent Ad infections. There are a number of reports of persistent Ad shedding in individuals following primary infection [39,40,43–47] consistent with our results.

We established a persistent Ad infection model *in vitro* and demonstrated that IFNγ suppresses productive Ad replication in a manner dependent on this conserved E2F binding site (Fig 7). Relief of this repression by removal of IFN resulted in a transition from persistent to lytic Ad infection. A viral mutant that lacks the conserved E2F binding in the E1A enhancer region was resistant to the effects of IFNs and was unable to establish extended persistent infection *in vitro*. IFN signaling reduced the binding of the major E1A enhancer transactivator complex GABPα/β to the E1A enhancer *in vivo*. The E2F binding site is located immediately adjacent to the GABPα/β binding site in the E1A enhancer. It is possible that GABPα/β and E2F/Rb proteins compete for binding to the enhancer given the proximity of their binding sites. Alternatively, IFNs may regulate the binding of GABPα/β and E2F/Rb family proteins to the E1A enhancer through independent mechanisms. In additional to the E1A enhancer region, E2Fs bind to sites in the Ad5 E2 promoter [48]. In NHBEC and HDF-TERT cells, IFNs exhibited stronger inhibition of transcription from the E2A and E2B regions compared with other early regions (Fig 1F and 1G) consistent with the idea that E2Fs also directly negatively regulate E2 expression in addition to E1A. This effect also was observed in HDF-TERT-E1A cells (Fig 2E).

These results reveal a novel mechanism by which adenoviruses utilize IFN signaling to inhibit lytic virus replication and promote persistent infection. It is well established that IFNs fail to inhibit wild-type Ad replication in established cell lines [5–7]. The resistance of wild-type Ad to the effects of IFNs is due to multiple counteracting effects of viral gene products. Additionally, this is due to the nature of established cancer lines that contain alterations in different signaling pathways. The same experimental conditions were utilized in experiments that demonstrated significant effects of IFNs in normal human cells but a distinct lack of effect in an adenocarcinoma cell line (Fig 1). Given the association of IFN signaling with E2F/Rb family protein function shown in our studies, we attribute defects observed in IFN signaling in cancer cell lines likely due to perturbations in regulation of E2F/Rb family members since the E2F pathway is mutated in numerous cancer cells [29]. In previous experiments, IFNα caused a modest reduction in Ad5 E1A and DBP expression and a 5–10 fold reduction in viral replication and virus yield in Ad5-infected NHBEC [12]. The mechanism of IFNα activity was not determined. We attribute the moderate effects observed in these assays to the profound effects of IFNs in our studies to potential differences in cell populations and/or the amount of IFNα used to treat the cells (250U/ml in [12], 500 U/ml here).

PML-NBs have established intrinsic and IFN-induced activity against many herpesviruses [49,50] which prompted us to examine if resident PML-NB proteins PML, Daxx or Sp100 inhibited Ad replication in HDF-TERT cells plus or minus IFN signaling. These activities were knocked-down individually and combinatorially using shRNAs. Depletion of these proteins singly or in combination did not alter the inhibitory effect of IFNs on Ad replication in a significant manner (S2 Fig). There was some variation among the cell lines on Ad5 infection minus IFN that we attribute to clonal variation. The lack of effect of these proteins on Ad replication, plus or minus IFN signaling, in comparison to herpesviruses may reflect the nature of the viral genomes within the virus particle. Herpesviruses contain naked viral DNA that may be easily recognized by cellular pattern recognition receptors to trigger IFN signaling and suppress virus infection [35]. In contrast, the Ad genome is coated by a histone-like core protein that may protect the genome from such recognition [51]. Consistent with this idea, we previously showed that Ad core protein VII protects the viral genome from recognition by the DNA damage machinery [52]. Thus, Ad may be resistant to the effects of PML-NBs in specific contexts.

The murine protein p202, a HIN-200 family member, is an IFN-inducible gene product that represses gene expression via E2F4/DP1 in transient expression assays [53,54]. These results and the known role of IFI16 in regulating cellular proliferation via CKIs which impact E2F-Rb activity [36–38] prompted us to examine if the human p202 homolog, IFI16, is involved in IFN-mediated repression of Ad replication in HDF-TERT cells. Depletion of IFI16 using shRNAs did not abrogate the negative effect of IFNs on Ad E1A expression and viral DNA replication in HDF-TERT cells (S3 Fig). Murine p202 regulates the DNA binding activity of E2F4/DP1 [53] and we found no effect of IFNs on E2F DNA binding properties *in vitro* plus or minus IFNs (Fig 6A).

We found that HDF-TERT cells infected with wild-type Ad5 continued proliferating without lysis over 100 days of maintenance in the presence of IFNγ (Fig 7). Viral DNA replication was restricted and maintained at a steady state level (1,000–10,000 viral genomes/cell). This number is comparable to persistent Ad infection in the BJAB and Ramos B cell lines [41]. In contrast, HDF-TERT cells infected with wild-type Ad5 and treated with IFNα initially maintained persistent Ad infection but succumbed by day 45 (Fig 7A). It is not clear why cells treated with IFNα did not maintain a persistent infection. Ad5-infected HDF-TERT cells continuously produced low amounts of infectious virus in these experiments suggesting that Ad established a persistent infection, rather than a latent infection, in the presence of IFNs. We attempted to study the effect of IFNs on the properties of Ad infection in lymphocytes (Jurkat and PM1 T cells and BJAB B cells). Even though these cells responded to IFN signaling (STAT1 phosphorylation was detected 1 hr after IFN treatment, STAT1 expression was induced by 24 hr), IFNα and IFNγ only had a minimal effect on Ad5 replication. These cell lines were derived from leukemias and lymphomas and likely contain alterations in the E2F/Rb pathway.

E1A protein expression did not block IFN signaling in HDF-TERT cells in our experiments (Fig 2C and 2F) in contrast to the inhibitory effect of E1A on Jak/STAT signaling and IFN-induced gene expression in previous experiments ([8–10] and references therein). In all but one of these reports, established cancer cell lines were used and we believe that IFN signaling may be altered in these cells compared to normal human cells used in our experiments. E1A was shown to inhibit IFN signaling in primary human tracheal cells [55] but using 10-fold less IFNγ than used here.

This is the first study that probes the molecular basis of lytic versus persistent adenovirus infection. We established a persistent Ad infection model *in vitro* and demonstrated that IFNγ suppresses productive Ad replication in a manner dependent on a conserved E2F binding site in the E1A enhancer region. These results reveal a novel mechanism by which adenovirus utilizes IFN signaling to suppress virus replication and promote persistent infection, and demonstrate that the IFN-E2F/Rb axis plays a critical role in this process.

## Materials and Methods

### Cells

Normal human bronchial epithelial cells (NHBEC) were purchased from Lonza and maintained in Bronchial Epithelial Cell Growth Medium containing BulletKits (Lonza) according to manufacturer’s instructions. HDF-TERT cells [56] were kindly provided by Dr. Kathleen Rundell (Northwestern University, Chicago, IL) and maintained in Dulbecco’s Modified Eagle’s Medium (DMEM) containing 10% fetal bovine serum (HyClone Laboratories). A549 cells (ATCC) were grown in DMEM containing 10% bovine calf serum. 293FT cells (Life Technologies) were used for the generation of lentivirus stocks and were maintained in DMEM containing 10% fetal bovine serum according manufacturer’s instructions. 293-TP cells [57] were used to propagate mutant virus ΔTP-GFP and were maintained in DMEM containing 10% fetal bovine serum and 400 μg/ml G418. All cell growth media were supplemented with 100 U/ml penicillin and 100 μg/ml streptomycin. Cells were treated with 500U/ml IFNα or 1000 U/ml IFNγ. The Ad5 E1A coding region was inserted into lentiviral expression vector pLenti6/v5-D-TOPO (Life Technologies). A lentivirus stock was generated by cotransfection of pLenti-E1A plasmid with ViraPower packaging mix (Life Technologies) into 293FT cells. HDF-TERT cells were transduced with the E1A-expressing lentivirus according to the manufacturer’s instructions. Pools of E1A-expressing HDF-TERT cells were obtained after selection with 1μg/ml blasticidin.

### Viruses

The replication defective adenovirus, ΔTP-GFP, was generously provided by Dr. Jerry Schaack (University of Colorado, Denver, CO). With ΔTP-GFP, the Ad5 terminal protein coding region is disrupted by the GFP gene. Mutant viruses, *in*340, *in*340-A5, *in*340-B1, *dl*309-21, *dl*309-3 and *dl*309-317/358, were previously described [20,27]. *In*340-Δ2-CMV, *dl*309-273/371, and pTG3602 mutants 1 through 3 (mut1–mut3) were generated by PCR and recombination using parent viruses *in*340 [20], *dl*309 [58], and TG3602 [59]. *In*340-Δ2-CMV contains a deletion of the E1A enhancer region which was replaced by the CMV promoter/enhancer region; Ad5 packaging sequences were inserted at the right-end of the viral genome [20].

### Replication assay

HDF-TERT cells were incubated with IFNs or left untreated for 24 hr, followed by adenovirus infection at 37°C for 1 hr at the multiplicities of infection indicated in figure legends. Nuclear DNA and total cell DNA were purified at 6 and 48 hr post-infection, respectively, using a QIAGEN DNeasy Blood & Tissue Kit. Both viral and cellular genome copy numbers were determined by qPCR using primer pairs that recognize either the Ad5 genome or cellular GAPDH gene with DyNAmo HS SYBR Green qPCR Kit (Thermo). The relative viral copy numbers of each time point were normalized to GAPDH. The fold-increase of viral copy number was calculated by normalizing to input viral DNA in 6 hr post-infection samples. Relative viral replication efficiency in IFN-treated cells was presented as the relative value compared to untreated cells. In the case of infection in A549 and NHBEC cells, nuclear and total DNA was harvested at 2 and 24 hr post-infection, respectively.

### RT-qPCR

Total RNA from infected cells was isolated using a QIAGEN RNeasy kit. Equal amounts of RNA from each sample were used for synthesis of first-strand cDNA using SuperScript II reverse transcriptase (Life Technologies) and quantified by qPCR using primer pairs that recognize different Ad5 early mRNAs or cellular GAPDH mRNA with DyNAmo HS SYBR Green qPCR Kit (Thermo). The Pfaffl method of relative quantification was used to convert the resulting threshold cycle data for each sample to relative fold change information [60]. Viral mRNA levels were normalized to cellular GAPDH mRNA.

### Chromatin Immunoprecipitation (ChIP)

ChIP was performed as described previously [61] with modification. HDF-TERT cells were pre-treated with IFNs or left untreated for 24 hr, followed by Ad infection at 37°C for 1 hr at 200 virus particles/cell. At 18 hr post-infection, cells were cross-linked by adding serum free DMEM containing 1% formaldehyde and incubated at 37°C for 10 min. Cross-linking was quenched by the addition of glycine to a final concentration of 125 mM. Cells were harvested and cell pellets were resupended in 1 ml SDS lysis buffer (50 mM Tris-HCl, pH 8.0, 10 mM EDTA, 1% SDS, 1 ug/ml aprotinin, 1 ug/ml pepstatin, 1 mM phenylmethanesulfonyl fluorid) per 10^7^ cells, followed by incubation on ice for 10 min. Lysed cells were sonicated to yield chromatin fragments of 200–1000 bp. Cellular debris was removed by high speed centrifugation. Cell lysates containing 100 μg chromatin were diluted 10-fold using dilution buffer (20 mM Tris-HCl, pH 8.0, 140 mM NaCl, 1.2 mM EDTA, 0.01% SDS, 1.1% Triton X-100, plus protease inhibitors) and pre-cleared by incubation with protein A-agarose/salmon sperm DNA slurry (Millipore) for 1 hr at 4°C with rotation. Samples were clarified by centrifugation and pre-cleared lysates incubated with 10 μg of anti-GABPα, anti-Rb, anti-p107, or anti-HA antibody overnight at 4°C. Immune complexes were captured using protein A-agarose/salmon sperm DNA for 2 hr at 4°C with rotation and pelleted by centrifugation. Immunoprecipitates were washed once with low-salt wash buffer (20 mM Tris-HCl, pH 8.0, 150 mM NaCl, 2 mM EDTA, 0.1% SDS, 1% Triton X-100, plus protease inhibitors), once with high salt wash buffer (20 mM Tris-HCl, pH 8.0, 500 mM NaCl, 2 mM EDTA, 0.1% SDS, 1% Triton X-100, protease inhibitors), once with LiCl wash buffer (10 mM Tris-HCl, pH 8.0, 0.25 M LiCl, 1 mM EDTA, 1% SDS, 0.5% Triton X-100, 1% sodium deoxycholate, plus protease inhibitors), and twice with TE buffer (10 mM Tris-HCl, pH 8.0, 1 mM EDTA, plus protease inhibitors). Immune complexes were eluted using 100 mM NaHCO_3_, 1% SDS at room temperature. Formaldehyde cross-links were reversed with 50 mM Tris-HCl, pH 7.5, 200 mM NaCl, 10 mM EDTA, and 0.5 mg/ml proteinase K at 65°C overnight. DNA was recovered by standard phenol/chloroform extraction and ethanol precipitation and resuspended in 50 μl 10 mM Tris-HCl, pH 7.5, 1 mM EDTA. 2 μl of DNA sample was subjected to qPCR. One μg pre-cleared chromatin was used to measure DNA input levels. The fold-enrichment of specific DNA target was presented as percentage of input DNA.

### Statistical analysis

All numerical values represent mean ± sd. Each experiment was done in three replicates, and a representative replicate is shown for each blot. Statistical significance of the differences was calculated using student’s t-test.

## Supporting Information

S1 Materials and Methods Additional Materials and Methods. (PDF)

S1 Fig Analysis of the adenovirus life cycle in HDF-TERT cells. (A) HDF-TERT cells on glass coverslips were infected with *dl*309 at 1,000 virus particles/cell and fixed at the indicated time points. Immunofluorescence assay was carried out using antibodies against PML (green) and Ad5 protein VII (red). (B, C) HDF-TERT cells were infected with *dl*309 at 25 virus particles/cell. Nuclear DNA and total RNA were purified at various time points. Viral DNA levels were quantified by qPCR and normalized to GAPDH DNA levels. Viral mRNA levels were quantified by RT-qPCR and normalized to GAPDH mRNA levels. (D) HDF-TERT cells were infected with *dl*309 at 200 virus particles/cell and harvested at 12, 16, 20 and 24 hr post-infection. Western blots were performed to examine viral early (E4-ORF3) and intermediate (IVa2) protein expression. γ-tubulin was used as a loading control for the samples. (E) HDF-TERT cells were pretreated with IFNs or left untreated, and then infected with *dl*309 at 25 virus particles/cell. Cells were harvested at 48, 97 and 144 hr post-infection and viral late protein levels (Penton, Hexon) were analyzed by Western blot. γ-tubulin was used as a loading control for the samples. (F) HDF-TERT cells on glass coverslips were pretreated with IFNα, ΙFNγ or left untreated for 24 hr, then infected with *dl*309 at 1,000 particles/cell. Cells were fixed at 2 hr post-infection. Immunofluorescence assays were carried out using antibodies against PML (green) and Ad5 protein VII (red).
(TIF)

S2 Fig Comparison of E1A expression levels. (A) Whole cell extracts were prepared from HDF-TERT cells (lane 1), HDF-TERT-E1A cells (lane 2), HDF-TERT cells infected with *dl*309 at 200 virus particles/cell and harvested at 48 hr post-infection (lane 3), and HDF-TERT cells infected with *in*340-Δ2-CMV-E1A (lane 4) or Δ1-3-CMV-12SHA (lane 5) at 5,000 virus particles/cells and harvested at 24 hr post-infection. E1A and γ-Tubulin levels were analyzed by Western blot. (B) HDF-TERT cells were infected with Ad5-WT, Ad5-mut1 or Ad5-mut2 virus at 25 particles/cell. Cells were harvested at 24 and 48 hr post-infection. E1A mRNA levels were analyzed by RT-qPCR and normalized to GAPDH. (C) HDF-TERT cells were infected with Ad5-WT at 1,000 particles/cell. Cells were harvested at 48 hr post-infection. E1A mRNA levels were analyzed by RT-qPCR and normalized to GAPDH.
(TIF)

S3 Fig Depletion of PML-NB components does not rescue Ad5 replication in the presence of IFNs. (A) Expression of PML, Daxx and Sp100 in single knockdown cell lines was examined by Western blot. (Left panel) Parental HDF-TERT cells (HDF) were used along with different individual knockdown subclones corresponding to control shRNA (shleer) and shRNAs targeting Daxx, PML and Sp100. (Right panel) Parental HDF-TERT cells (HDF) were used along with pools of cells expressing control shRNA (shneg) and shRNAs targeting Daxx and PML (shDP), PML and Sp100 (shPS), and all three proteins (shDPS). (B) Single-knockdown cell lines (left panel) and double- and triple-knockdown cell pools (right panel) were pretreated with IFNs or left untreated for 24 hr and then infected with *dl*309 at 1,000 virus particles/cell. Viral DNA replication was quantified by qPCR at 48 hr post-infection and was plotted as mean ± sd, n = 4. (C) HDF-TERT cells were infected with *dl*309 at 1,000 particles/cell. Cells were harvested at 48 hr post-infection. E1A mRNA levels were analyzed by RT-qPCR and normalized to GAPDH.
(TIF)

S4 Fig Replication of Ad5 in IFI16-depleted cells. (A) IFI16 proteins levels were examined by Western blot in parental HDF-TERT cells (HDF) along with different individual knockdown subclones corresponding to control shRNA (shneg) and shRNAs targeting IFI16. γ-tubulin was used as a loading control for the samples. (B) shRNA control and IFI16 knockdown cells were treated with IFNs or left untreated for 48 hr. IFI16 protein levels were examined by Western blot. (C) shRNA control and IFI16 knockdown cells were pretreated with IFNs or left untreated and then infected with *dl*309 at 1,000 virus particles/cell. Viral DNA replication was quantified by qPCR at 48 hr post-infection and was plotted as mean ± sd.
(TIF)

S5 Fig Analyses of HDF-TERT long-term Ad infections. (A) Phase contrast images were captured from HDF-TERT cells infected with Ad5-WT or Ad5-mut1, with or without IFNγ, as described in Fig 7 at the indicated days post-infection. (B) E1A expression was analyzed with HDF-TERT cells infected with Ad5-WT or Ad5-mut1 by Western blot at the indicated time points. α-Tubulin was used as a loading control.
(TIF)
