# Supplementary material for: E2F/Rb Family Proteins Mediate Interferon Induced Repression of Adenovirus Immediate Early Transcription to Promote Persistent Viral Infection
Source: PLoS Pathog. 2016 Jan 25;12(1):e1005415. doi: 10.1371/journal.ppat.1005415 (PMC4726734; doi:10.1371/journal.ppat.1005415)
Supplement: S1 Materials and Methods — (PDF) [file ppat.1005415.s001.pdf]

Supplemental Information

Materials and Methods

Antibodies and Reagents

For detection of viral proteins, the following antibodies were used: anti-E1A (sc-430, Santa Cruz Biotechnology), anti-DBP (clone B6-8)[1], anti-E4ORF3 (clone 6A11)[2], anti-E1B55K (clone 2A6)[3], anti-pVII [4], anti-Hexon (sc-58085, Santa Cruz Biotechnology, and 65H6, Abnova), anti-Penton (gift of Dr. Carl Anderson, Brookhaven National Laboratory, Upton, NY). For detection of PML-NB components, anti-PML (H-238, Santa Cruz Biotechnology), anti-Daxx (25C12, Cell Signaling Technology) and anti-Sp100 (AB1380, Chemicon International) antibodies were used. For EMSA antibody supershift reactions, anti-E2F-3 (C-20), anti-E2F-4 (C-20 and C-108), anti-E2F-5 (C-20), anti-Rb (C-15), anti-p107 (C-108 and SD9X), anti-p130 (C-20X) and anti-DP-1 (K-20) were from Santa Cruz Biotechnology and anti-E2F-1 (KH95) was from Lab Vision/NeoMarkerr. For immunoprecipitation and chromatin immunoprecipitation reactions, anti-GABPα (H-180), anti-GABPβ (E-7), anti-Rb (C-15), and anti-p107 (C-108) were from Santa Cruz Biotechnology and anti-HA was from Rockland Immunochemicals. Human IFNα (universal type I interferon 11200-2) and human IFNγ (11500-2) were from PBL Assay Science, dissolved in phosphate buffered saline (PBS) containing 0.1% BSA according to the manufacturer’s instructions. PD0332991 (Palbociclib) was from APExBIO and dissolved in DMSO.

Immunoprecipitation and immunofluorescence assays

HDF-TERT cells were treated with IFNs or left untreated for 24 hr, and immunoprecipitation assays were carried out as described previously [5]. Immunoprecipitations were performed using 2 μg GABPα antibody with protein A-agarose and analyzed by western blot using an antibody against GABPβ. HDF-TERT

cells were seeded on glass coverslips and infected as described above. At appropriate time points, the cells were fixed with ice-cold methanol and processed for immunofluorescence as described [6]. Images were captured and analyzed using a Zeiss Axiovert 200M digital deconvolution microscope with AxioVision 4.8.2 SP3 software.

Electrophoretic mobility shift assays (EMSA)

HDF-TERT cells were incubated with IFNs or left untreated for 24 hr, and nuclear extracts were prepared as described previously [7]. EMSA binding reactions were performed in a total volume of 15 μl containing 20 mM HEPES, pH 7.9, 5 mM $MgCl_2$, 50 mM KCl, 1 mM DTT, and 6% glycerol. 4 μg of each nuclear extract was incubated with 250 ng sonicated salmon sperm DNA, with or without 0.2 μg specific antibody, for 15 min at room temperature. 10 fmol (80,000 cpm) of ^{32}P-labeled probe was added to each reaction and incubated for an additional 30 min at room temperature. DNA-protein complexes were resolved in a native polyacrylamide gel. The gels were dried and autoradiographed. The E1A-ENH probe, 5'-GGTTCCATTTTCGCGGGAAAACTGCCGC-3', corresponds to Ad5 nt 271-288.

Generation of shRNA knockdown cell ines

For individual depletion of PML, Daxx or Sp100, retroviral vectors expressing shRNAs directed against PML, Daxx and Sp100 (pSIREN-RetroQ-shPML/-shDaxx/-shSp100), as well as negative control pSIREN-RetroQ-shLuci, were used [8,9]. Replication-deficient retroviruses were generated by cotransfection of 293FT cells (Life Technologies) with helper virus and pVSV-G in combination with the respective pSIREN-RetroQ plasmids. Retrovirus-containing medium was repeatedly collected at 2 days, 3 days and 4 days post-transfection. For double and triple depletion of PML-NB components, lentiviral vectors, pLKO-shDP/shPS/shDPS, as well as a scrambled negative control, pLKO-shneg were used [10]. Lentivirus stocks were prepared as

previously described [10]. To deplete endogenous IFI16 protein, scrambled shRNA in pLKO-shneg was replaced with shRNA against IFI16. shRNA target sequences against IFI16 were obtained from the RNAi consortium (TRC) shRNA library, siIFI16-1, GATCATTGCCATAGCAAATT; siIFI16-3, GGAAACTCTGAAGATTGATT. Low passage HDF-TERT cells were transduced with the retrovirus or lentivirus supernatants in the presence of 7.5 μg/ml polybrene (Sigma-Aldrich, Germany) overnight. To increase the transduction efficiency, HDF-TERT cells were reinfected with retroviruses or lentiviruses at day 2 and day 3. Stably-transduced pools of cell populations were selected using 2 μg/ml puromycin. At least two independent pools of cells with efficient knowdown of individual target genes were generated and analyzed.

Western blot analysis

Whole cell extracts were prepared by suspending cell pellets in SDS lysis buffer (50 mM Tris-HCl, pH 6.5, 4% SDS) and boiled for 10 min. The samples were clarified by centrifugation and the protein concentration was determined using a Bicinchoninic Acid (BCA) kit (Pierce). Equal amounts of whole cell extracts were resolved on SDS-PAGE and transferred to a nitrocellulose membrane. Membranes were blocked with Tris-HCl-buffered saline (TBS) buffer containing 3% BSA for 1 hr at room temperature, followed by incubation with primary antibodies (as indicated in the text and figure legends) at 4°C overnight. Membranes were washed with TBS buffer containing 0.1% Tween-20 (TBS-T) and then incubated with IRDye® 800CW-conjugated goat anti-rabbit antibody (926-32211, Li-COR) and IRDye® 680RD-conjugated goat anti-mouse antibody (925-068071, Li-COR) for 1 hr at room temperature. After three wash with TBS-T, images was captured using the ODYSSEY® CLx infrared imaging system (Li-COR). Alternatively, HRP-conjugated secondary antibodies were used in conjunction with ECL western blotting (Millipore Immobilon).

Cell fractionation

For the preparation of isolated nuclei for qPCR, cells were washed twice with ice-cold PBS, harvested, resuspended in 1 ml ice-cold isotonic buffer containing NP-40 (10 mM Tris-HCl, pH 7.5, 140 mM NaCl, 1.5 mM $MgCl_2$, 0.6% NP-40), and incubated on ice for 10 min. Nuclei were pelleted by centrifugation at 2,000 x g for 10 min at 4 °C. Nuclear and cytoplasmic fractions for Western blot analysis were prepared using NE-PER™ nuclear and cytoplasmic extraction kit (Pierce).

Figure Legends

Figure S1. Analysis of the adenovirus life cycle in HDF-TERT cells. (A) HDF-TERT cells on glass coverslips were infected with *dl*309 at 1,000 virus particles/cell and fixed at the indicated time points. Immunofluorescence assay was carried out using antibodies against PML (green) and Ad5 protein VII (red). (B, C) HDF-TERT cells were infected with *dl*309 at 25 virus particles/cell. Nuclear DNA and total RNA were purified at various time points. Viral DNA levels were quantified by qPCR and normalized to GAPDH DNA levels. Viral mRNA levels were quantified by RT-qPCR and normalized to GAPDH mRNA levels. (D) HDF-TERT cells were infected with *dl*309 at 200 virus particles/cell and harvested at 12, 16, 20 and 24 hr post-infection. Western blots were performed to examine viral early (E4-ORF3) and intermediate (IVa2) protein expression. γ-tubulin was used as a loading control for the samples. (E) HDF-TERT cells were pretreated with IFNs or left untreated, and then infected with *dl*309 at 25 virus particles/cell. Cells were harvested at 48, 97 and 144 hr post-infection and viral late protein levels (Penton, Hexon) were analyzed by Western blot. γ-tubulin was used as a loading control for the samples. (F) HDF-TERT cells on glass coverslips were pretreated with IFNα, IFNγ or left untreated for 24 hr, then infected with *dl*309 at 1,000 particles/cell. Cells were fixed at 2 hr post-infection. Immunofluorescence assays were carried out using antibodies against PML (green) and Ad5 protein VII (red).

Figure S2. Comparison of E1A expression levels. (A) Whole cell extracts were prepared from HDF-TERT cells (lane 1), HDF-TERT-E1A cells (lane 2), HDF-TERT cells infected with *dl*309 at 200 virus particles/cell and harvested at 48 hr post-infection (lane 3), and HDF-TERT cells infected with *in*340-Δ2-CMV-E1A (lane 4) or Δ1-3-CMV-12SHA (lane 5) at 5,000 virus particles/cells and harvested at 24 hr post-infection. E1A and γ-Tubulin levels were analyzed by Western blot. (B) HDF-TERT cells were infected with Ad5-WT, Ad5-mut1 or Ad5-mut2 virus at 25 particles/cell. Cells were harvested at 24 and 48 hr post-infection. E1A mRNA levels were analyzed by RT-qPCR and normalized to GAPDH. (C) HDF-TERT cells were infected with Ad5-WT at 1,000 particles/cell. Cells were harvested at 48 hr post-infection. E1A mRNA levels were analyzed by RT-qPCR and normalized to GAPDH.

Figure S3. Depletion of PML-NB components does not rescue Ad5 replication in the presence of IFNs. (A) Expression of PML, Daxx and Sp100 in single knockdown cell lines was examined by Western blot. (Left panel) Parental HDF-TERT cells (HDF) were used along with different individual knockdown subclones corresponding to control shRNA (shleer) and shRNAs targeting Daxx, PML and Sp100. (Right panel) Parental HDF-TERT cells (HDF) were used along with pools of cells expressing control shRNA (shneg) and shRNAs targeting Daxx and PML (shDP), PML and Sp100 (shPS), and all thre proteins (shDPS). (B) Single-knockdown cell lines (left panel) and double- and triple-knockdown cell pools (right panel) were pretreated with IFNs or left untreated for 24 hr and then infected with *dl*309 at 1,000 virus particles/cell. Viral DNA replication was quantified by qPCR at 48 hr post-infection and was plotted as mean ± sd, n=4. (C) HDF-TERT cells were infected with *dl*309 at 1,000 particles/cell. Cells were harvested at 48 hr post-infection. E1A mRNA levels were analyzed by RT-qPCR and normalized to GAPDH.

Figure S4. Replication of Ad5 in IFI16-depleted cells. (A) IFI16 proteins levels were examined by Western blot in parental HDF-TERT cells (HDF) along with different individual knockdown subclones corresponding to control shRNA (shneg) and shRNAs targeting IFI16. γ-tubulin was used as a loading control for the samples. (B) shRNA control and IFI16 knockdown cells were treated with IFNs or left untreated for 48 hr. IFI16 protein levels were examined by Western blot. (C) shRNA control and IFI16 knockdown cells were pretreated with IFNs or left untreated and then infected with *dl*309 at 1,000 virus particles/cell. Viral DNA replication was quantified by qPCR at 48 hr post-infection and was plotted as mean ± sd.

Figure S5. Analyses of HDF-TERT long-term Ad infections. (A) Phase contrast images were captured from HDF-TERT cells infected with Ad5-WT or Ad5-mut1, with or without IFNγ, as described in Figure 7 at the indicated days post-infection. (B) E1A expression was analyzed with HDF-TERT cells infected with Ad5-WT or Ad5-mut1 by Western blot at the indicated time points. α-Tubulin was used as a loading control.

Table S1. Oligonucleotides used in qPCR.

PACK-4	GCGAAAATGGCCAAATGTTA	Left-end of Ad5-WT, spanning from the ITR to the E1A enhancer region
PACK-5	TAATGAGGGGGTGGAGTTTG	
loxP7	GTCCGGTTTCTATGCCAAAC	E1A 13S cDNA, does not recognize Ad-CMV and Ad-CMV-12SHA
loxP8	CCGTATTCCTCCGGTGATAA	
E1B-F	TGTGCCTTTTACTGCTGCTG	E1B cDNA
E1B-R	CACAGCCACGCTTTTCACTA	
DBP-F	CCGTAGTGGCATCAAAAGGT	E2A cDNA
DBP-R	GTCTAGCAAGGCCAAGATCG	
E2B-F	CGCGCGTCGAAGTAGTCTAT	E2B cDNA
E2B-R	CGGTGGAAGATGCTACCCTA	
E4ORF6-F	TACCGGGAGGTGGTGAATTA	E4ORF6 cDNA
E4ORF6-R	TTCAAAATCCCACAGTGCAA	
E1A-ENH-F	CGCGGGAAAACTGAATAAGA	Ad5-WT and *dl*309-specific, does not recognize *in*340-Δ2-CMV
E1A-ENH-R	CTTGAGGAACTCACCGGGTA	
Ad5-mut1-F	ATATCTCCCACTGAATAAGA	Ad5-mut1 E1A enhancer-specific
E1A-pan-F	ACTCTTGAGTGCCAGCGAG	
Ad5E1A-R	ACTCTTGAGTGCCAGCGAG	
Ad3E1A-R	TACAGATCGTGCAGCGTAGG	
Ad4-E1A-R	AGCGAAGGTGTCTCAAATGG	
Ad9-E1A-R	GGGCATCTACCTCCAGATCA	
Ad12-E1A-R	CGGCAGACTCCACATCAAG	
cGAPDH-F	ACCCAGAAGACTGTGGATGG	cellular GAPDH cDNA
cGAPDH-R	TTCTAGACGGCAGGTCAGGT	
gGAPDH-F	CCCCACACACATGCACTTACC	cellular GAPDH genomic sequence
gGAPDH-R	CCTAGTCCCAGGGCTTTGATT	

References

1. Reich NC, Sarnow P, Duprey E, Levine AJ (1983) Monoclonal antibodies which recognize native and denatured forms of the adenovirus DNA-binding protein. Virology 128: 480-484.
2. Nevels M, Tauber B, Kremmer E, Spruss T, Wolf H, et al. (1999) Transforming potential of the adenovirus type 5 E4orf3 protein. J Virol 73: 1591-1600.
3. Sarnow P, Sullivan CA, Levine AJ (1982) A monoclonal antibody detecting the adenovirus type 5-E1b-58Kd tumor antigen: characterization of the E1b-58Kd tumor antigen in adenovirus-infected and -transformed cells. Virology 120: 510-517.
4. Chen J, Morral N, Engel DA (2007) Transcription releases protein VII from adenovirus chromatin. Virology 369: 411-422.
5. Sohn SY, Hearing P (2011) Adenovirus Sequesters Phosphorylated STAT1 at Viral Replication Centers and Inhibits STAT Dephosphorylation. J Virol 85: 7555-7562.
6. Karen KA, Hearing P (2011) Adenovirus core protein VII protects the viral genome from a DNA damage response at early times after infection. J Virol 85: 4135-4142.
7. Guimet D, Hearing P (2013) The adenovirus L4-22K protein has distinct functions in the posttranscriptional regulation of gene expression and encapsidation of the viral genome. J Virol 87: 7688-7699.
8. Tavalai N, Adler M, Scherer M, Riedl Y, Stamminger T (2011) Evidence for a dual antiviral role of the major nuclear domain 10 component Sp100 during the immediate-early and late phases of the human cytomegalovirus replication cycle. J Virol 85: 9447-9458.
9. Tavalai N, Papior P, Rechter S, Stamminger T (2008) Nuclear domain 10 components promyelocytic leukemia protein and hDaxx independently contribute to an

intrinsic antiviral defense against human cytomegalovirus infection. J Virol 82: 126-137.

10. Glass M, Everett RD (2012) Components of PML Nuclear Bodies (ND10) act cooperatively to repress herpesvirus infection. J Virol 87: 2174-2185.
