# Supplementary figures and images for: E2F/Rb Family Proteins Mediate Interferon Induced Repression of Adenovirus Immediate Early Transcription to Promote Persistent Viral Infection
Source: PLoS Pathog. 2016 Jan 25;12(1):e1005415. doi: 10.1371/journal.ppat.1005415 (PMC4726734; doi:10.1371/journal.ppat.1005415)

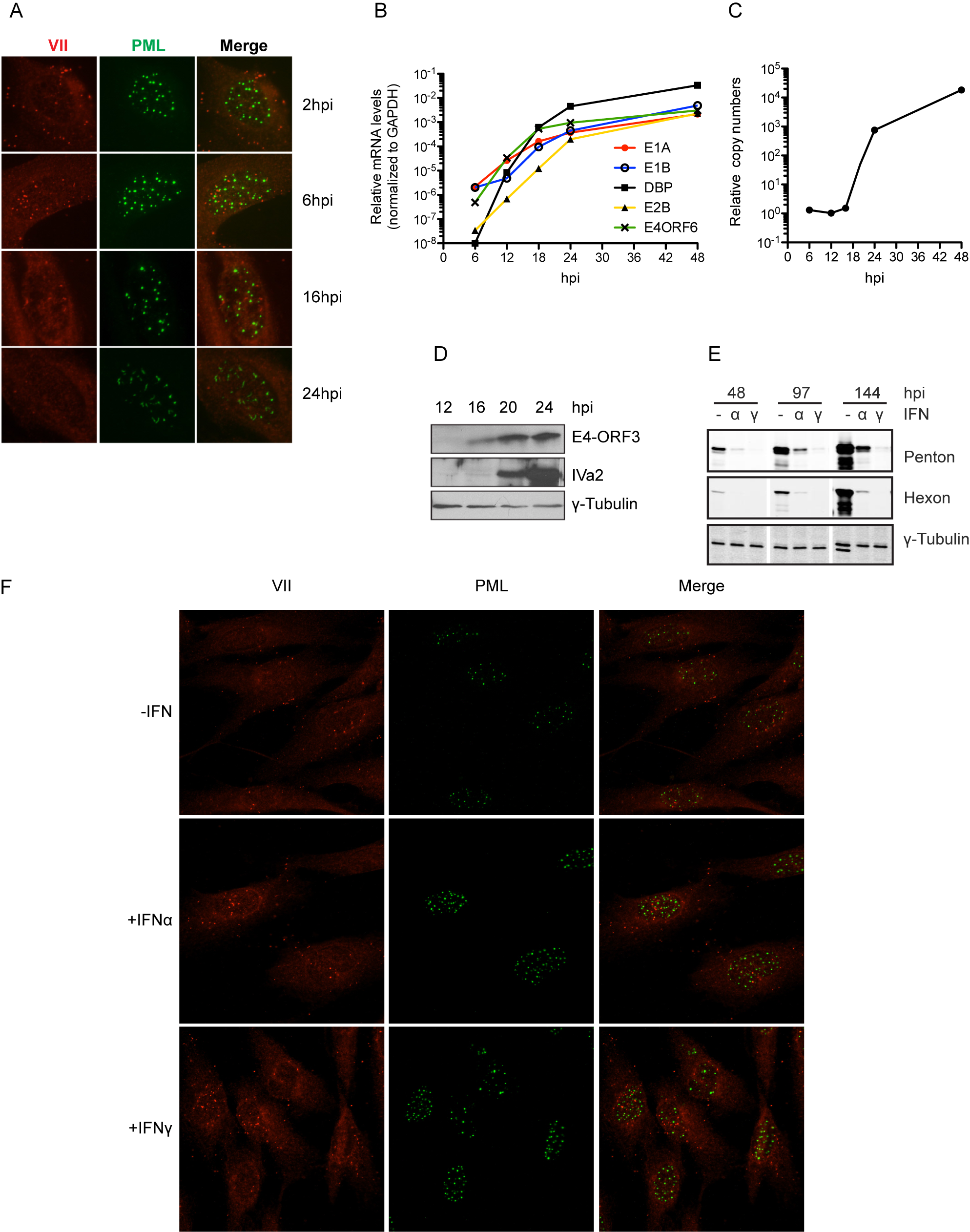

Supplement: S1 Fig — (A) HDF-TERT cells on glass coverslips were infected with dl309 at 1,000 virus particles/cell and fixed at the indicated time points. Immunofluorescence assay was carried out using antibodies against PML (green) and Ad5 protein VII (red). (B, C) HDF-TERT cells were infected with dl309 at 25 virus particles/cell. Nuclear DNA and total RNA were purified at various time points. Viral DNA levels were quantified by qPCR and normalized to GAPDH DNA levels. Viral mRNA levels were quantified by RT-qPCR and normalized to GAPDH mRNA levels. (D) HDF-TERT cells were infected with dl309 at 200 virus particles/cell and harvested at 12, 16, 20 and 24 hr post-infection. Western blots were performed to examine viral early (E4-ORF3) and intermediate (IVa2) protein expression. γ-tubulin was used as a loading control for the samples. (E) HDF-TERT cells were pretreated with IFNs or left untreated, and then infected with dl309 at 25 virus particles/cell. Cells were harvested at 48, 97 and 144 hr post-infection and viral late protein levels (Penton, Hexon) were analyzed by Western blot. γ-tubulin was used as a loading control for the samples. (F) HDF-TERT cells on glass coverslips were pretreated with IFNα, ΙFNγ or left untreated for 24 hr, then infected with dl309 at 1,000 particles/cell. Cells were fixed at 2 hr post-infection. Immunofluorescence assays were carried out using antibodies against PML (green) and Ad5 protein VII (red). (TIF) [file ppat.1005415.s002.tif]

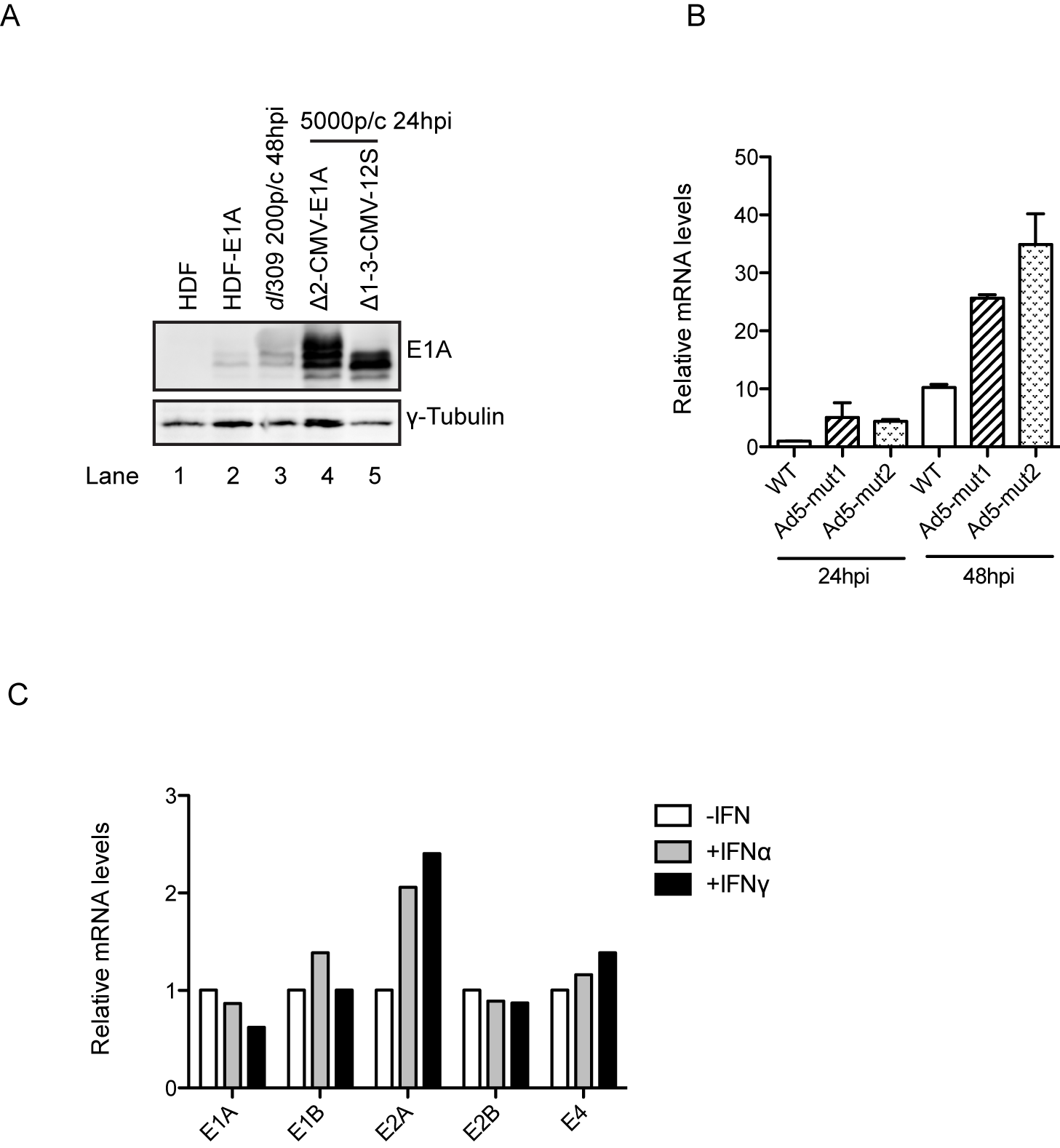

Supplement: S2 Fig — (A) Whole cell extracts were prepared from HDF-TERT cells (lane 1), HDF-TERT-E1A cells (lane 2), HDF-TERT cells infected with dl309 at 200 virus particles/cell and harvested at 48 hr post-infection (lane 3), and HDF-TERT cells infected with in340-Δ2-CMV-E1A (lane 4) or Δ1-3-CMV-12SHA (lane 5) at 5,000 virus particles/cells and harvested at 24 hr post-infection. E1A and γ-Tubulin levels were analyzed by Western blot. (B) HDF-TERT cells were infected with Ad5-WT, Ad5-mut1 or Ad5-mut2 virus at 25 particles/cell. Cells were harvested at 24 and 48 hr post-infection. E1A mRNA levels were analyzed by RT-qPCR and normalized to GAPDH. (C) HDF-TERT cells were infected with Ad5-WT at 1,000 particles/cell. Cells were harvested at 48 hr post-infection. E1A mRNA levels were analyzed by RT-qPCR and normalized to GAPDH. (TIF) [file ppat.1005415.s003.tif]

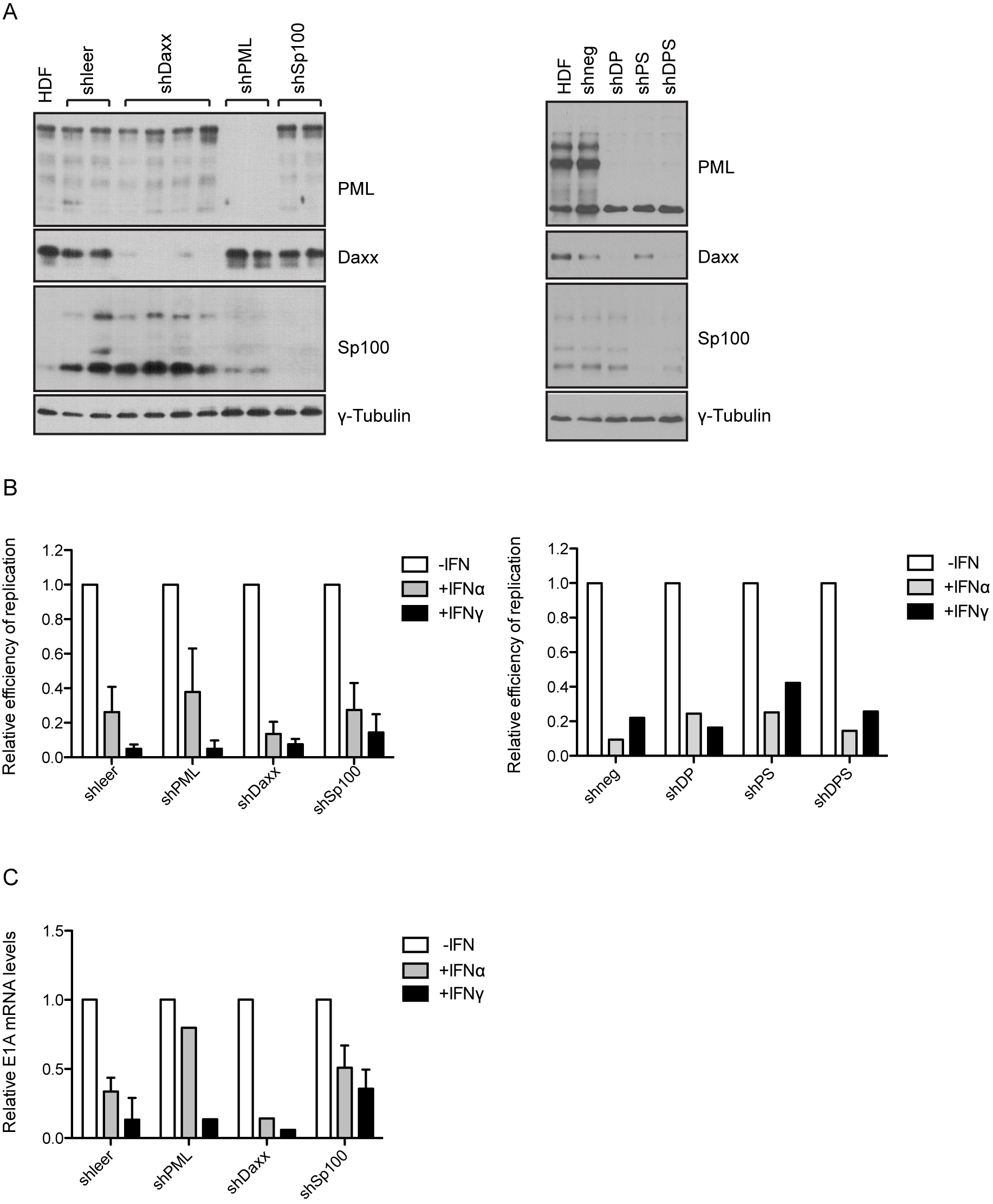

Supplement: S3 Fig — (A) Expression of PML, Daxx and Sp100 in single knockdown cell lines was examined by Western blot. (Left panel) Parental HDF-TERT cells (HDF) were used along with different individual knockdown subclones corresponding to control shRNA (shleer) and shRNAs targeting Daxx, PML and Sp100. (Right panel) Parental HDF-TERT cells (HDF) were used along with pools of cells expressing control shRNA (shneg) and shRNAs targeting Daxx and PML (shDP), PML and Sp100 (shPS), and all three proteins (shDPS). (B) Single-knockdown cell lines (left panel) and double- and triple-knockdown cell pools (right panel) were pretreated with IFNs or left untreated for 24 hr and then infected with dl309 at 1,000 virus particles/cell. Viral DNA replication was quantified by qPCR at 48 hr post-infection and was plotted as mean ± sd, n = 4. (C) HDF-TERT cells were infected with dl309 at 1,000 particles/cell. Cells were harvested at 48 hr post-infection. E1A mRNA levels were analyzed by RT-qPCR and normalized to GAPDH. (TIF) [file ppat.1005415.s004.tif]

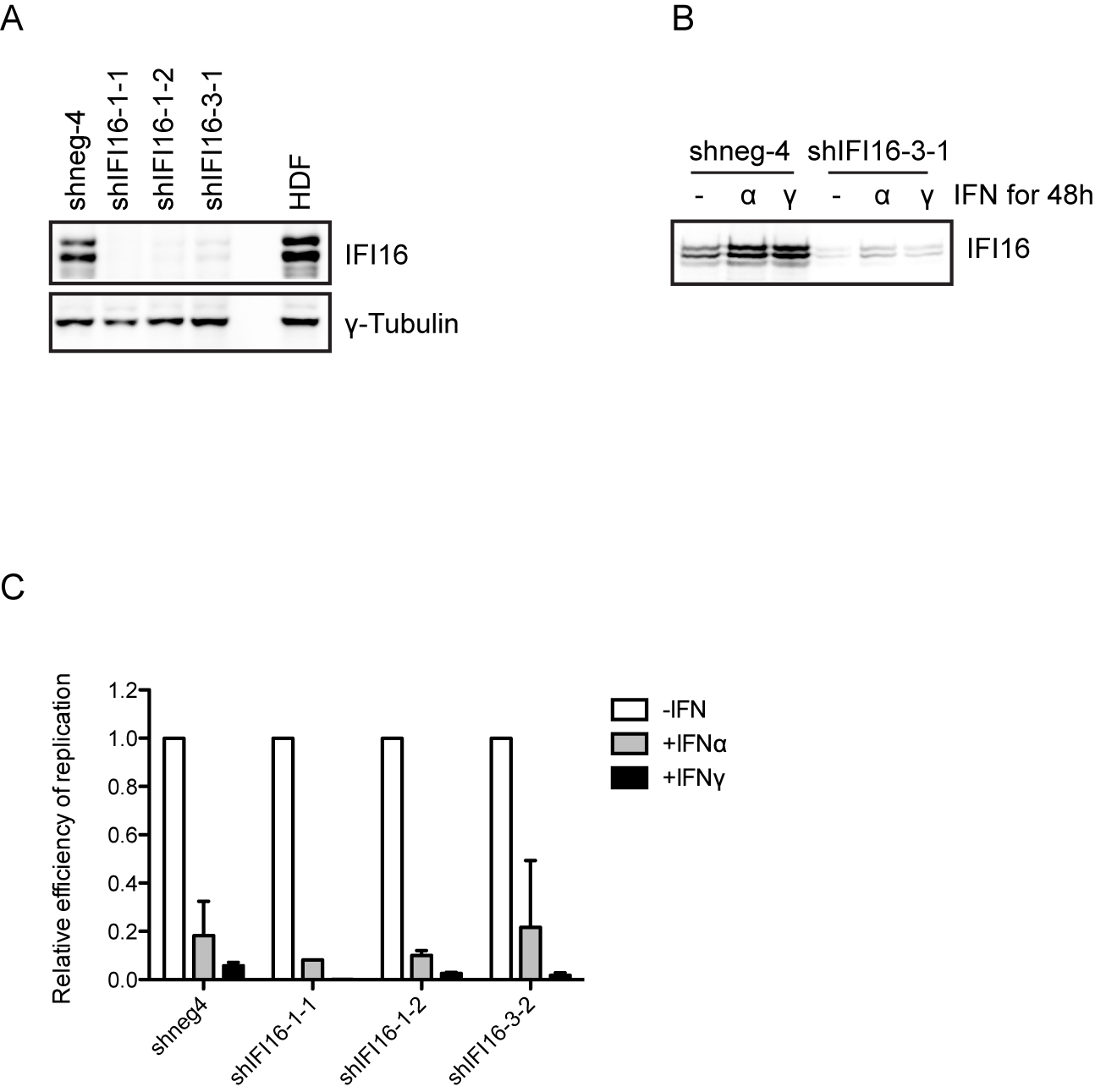

Supplement: S4 Fig — (A) IFI16 proteins levels were examined by Western blot in parental HDF-TERT cells (HDF) along with different individual knockdown subclones corresponding to control shRNA (shneg) and shRNAs targeting IFI16. γ-tubulin was used as a loading control for the samples. (B) shRNA control and IFI16 knockdown cells were treated with IFNs or left untreated for 48 hr. IFI16 protein levels were examined by Western blot. (C) shRNA control and IFI16 knockdown cells were pretreated with IFNs or left untreated and then infected with dl309 at 1,000 virus particles/cell. Viral DNA replication was quantified by qPCR at 48 hr post-infection and was plotted as mean ± sd. (TIF) [file ppat.1005415.s005.tif]

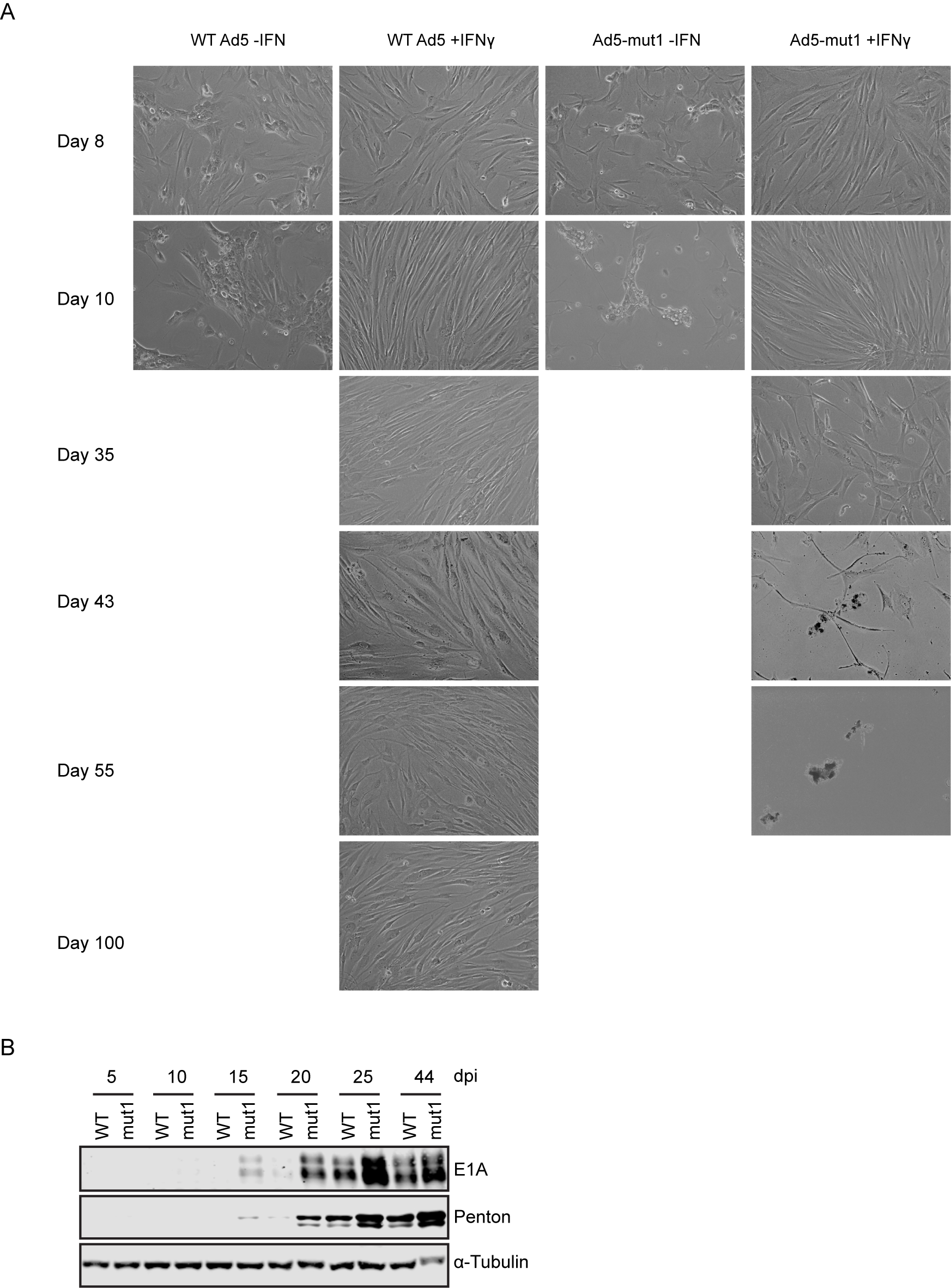

Supplement: S5 Fig — (A) Phase contrast images were captured from HDF-TERT cells infected with Ad5-WT or Ad5-mut1, with or without IFNγ, as described in Fig 7 at the indicated days post-infection. (B) E1A expression was analyzed with HDF-TERT cells infected with Ad5-WT or Ad5-mut1 by Western blot at the indicated time points. α-Tubulin was used as a loading control. (TIF) [file ppat.1005415.s006.tif]
